# Governator vs. Hunter and Aggregator: A simulation of party competition with vote-seeking and office-seeking rules

**DOI:** 10.1371/journal.pone.0191649

**Published:** 2018-02-02

**Authors:** Roni Lehrer, Gijs Schumacher

**Affiliations:** 1 Collaborative Research Center SFB 884, University of Mannheim, Mannheim, Germany; 2 Department of Political Science, University of Amsterdam, Amsterdam, The Netherlands; University of Vermont, UNITED STATES

## Abstract

The policy positions parties choose are central to both attracting voters and forming coalition governments. How then should parties choose positions to best represent voters? Laver and Sergenti show that in an agent-based model with boundedly rational actors a decision rule (Aggregator) that takes the mean policy position of its supporters is the best rule to achieve high congruence between voter preferences and party positions. But this result only pertains to representation by the legislature, not representation by the government. To evaluate this we add a coalition formation procedure with boundedly rational parties to the Laver and Sergenti model of party competition. We also add two new decision rules that are sensitive to government formation outcomes rather than voter positions. We develop two simulations: a single-rule one in which parties with the same rule compete and an evolutionary simulation in which parties with different rules compete. In these simulations we analyze party behavior under a large number of different parameters that describe real-world variance in political parties’ motives and party system characteristics. Our most important conclusion is that Aggregators also produce the best match between government policy and voter preferences. Moreover, even though citizens often frown upon politicians’ interest in the prestige and rents that come with winning political office (office pay-offs), we find that citizens actually receive better representation by the government if politicians are motivated by these office pay-offs in contrast to politicians with ideological motivations (policy pay-offs). Finally, we show that while more parties are linked to better political representation, how parties choose policy positions affects political representation as well. Overall, we conclude that to understand variation in the quality of political representation scholars should look beyond electoral systems and take into account variation in party behavior as well.

## Introduction

How are citizens’ policy preferences optimally represented by political parties, by the legislature, and by the government? That is, under what circumstances do they suggest and implement policies that their voters like? First answers to these questions were developed in spatial models of politics with rational actors [1], which are based on location theory [2,3]. Simulation evidence with boundedly rational actors suggests that when all political parties take the mean policy position of their voters, optimal representation of voter preferences in the legislature will eventually be achieved [4]. This result falls apart when parties behave differently: for example when parties stick to their position, shift to the position of other successful parties, or shift away from positions that brought electoral losses. These insights stem from an agent-based model of elections developed by Laver and Sergenti [4–7]. Their model is “about vote shares at legislative elections” [4]and a coalition formation process is missing. In virtually all models of party politics, however, parties ultimately care about realizing policies which they favor for ideological reasons (policy) or the prestige associated with public office, the rents and income that can be extracted (office). Votes are only an instrument to achieve these other goals [8]. For most parliamentary political parties policy influence and office pay-offs are achieved through coalition formation processes. In this paper we extend the Laver and Sergenti agent-based model by adding a boundedly rational coalition formation process. Thus, we can evaluate the effect of coalition formation on citizen representation. Also, we can evaluate what behavior of political parties fosters optimal representation of citizens by the government.

Why do we simulate? Following Laver and Sergenti [4] politics is dynamic and complex, and politicians are diverse. Even though parties invest in research on voters and their opponents, party strategies often fail due to the high uncertainty and complexity of party competition [7,9]. Hence parties do not know what policy shifts lead to an increase in their vote share or increase influence on government formation. We argue that this uncertainty carries over to government formation as well. Agent-based models are invaluable tools to develop predictions in complex systems with adaptive and diverse actors [10,11]–such as political competition in a multi-dimensional context [4,12–17]. In this paper, the agent-based model produces outcomes of political systems in which parties with different goals, decision-making rules and positions compete. Such complexity easily becomes intractable with game-theoretic methods. Moreover, by simulating agent-based models we can consider a whole range of scenarios and their consequences for representation that do not occur in “nature”.

How do we simulate? We start from the agent-based model developed by Laver and Sergenti [4–7]. The basic premise of this model is that parties cannot predict what electoral consequences policy shifts have and therefore cannot derive strategies that maximize future performance. Instead parties use heuristics, i.e. rules of thumb, to make decisions about strategy. For example, the Hunter rule [7] repeats a policy shift in the same direction if it won votes in the previous election, but shifts in the opposite direction if it lost votes. Other examples of party rules are Aggregator (move to the mean of party supporters at previous election) or Sticker (stay put). In each round of our simulation, parties take position following their assigned rule, an election is held with voters voting for the party closest to them, and parties subsequently form a coalition government (if no party attained a majority). *The boundedly rational coalition formation model is the first innovation* compared to the Laver and Sergenti model that enables us to also analyze representation by the government [18]. To our knowledge, it is the first government formation model with boundedly rational actors [13,19,20]. We simulate a very large number of models with different parameterizations of the degree to which parties value office or policy pay-offs, the number of parties in the simulation, parties using different rules, characteristics of the electorate, discounting of caretaker government utility, party aspiration and party memory of past results. We use these different model parameterizations to predict which party system produces optimal representation in terms of how parties and governments represent voters.

Which rules compete in our simulation? With the exception of the Sticker, the rules developed by Laver and Sergenti [4] are primarily responsive to voters, they are vote-oriented. The Hunter responds to changes in its vote share and the Aggregator moves to the mean position of its party supporters. The Hunter rule is a so-called hill-climbing algorithm and is good at finding high concentrations of voters, and thus performs rather well in terms of representation of voter preferences [7]. Parties using the Aggregator rule spread out over the distribution of voter preferences. This way they become more eccentric, but also maximize congruence between parties and voters [4]. *The second innovation is the addition of rules that orient toward the government policy position*. We introduce the Governator and Satisficing Governator rules. The Governator moves at each election towards the position of the outgoing government. The Satisficing Governator does the same as the Governator, but stays put once in government. By letting rules responsive to governing compete with rules responsive to voters we address an important issue in the formal and empirical study of party competition. It is still very often assumed that parties are responsive to voters [3], and there is little research regarding how and to what extent parties would respond to the main prize of political competition: winning or losing government office [21]. Our simulations allow for a comparison of how vote-oriented and office-oriented rules perform in terms of representation.

We present simulated data from two different simulations. In the first simulation we only let parties with the same rule compete under a large number of different model parameters. In the second simulation we let all rules compete with each other and we add an evolutionary component so that underperforming parties randomly select other decision rules. This way we can develop predictions regarding which rules we are likely to observe in nature.

Our model produces three hypotheses about representation of voter preferences that can be tested by empirical research. First, Aggregator behavior is best for representation. Second, policy-seeking behavior surprisingly depresses representation and, third, more parties are better for representation, yet individual party behavior mitigates this effect. In the following, we describe the model we use to simulate party systems (including government formation and parties’ decision rules). Obtaining meaningful results from the model is, unfortunately, not straightforward, and hence, we discuss our simulation strategy before turning to the interpretation of the single-rule simulations. Then, we introduce the evolutionary element. Finally, we summarize our results, suggest hypotheses that can be tested in empirical research and discuss real-world implications.

## The model

We develop an agent-based model including parties’ policy shifts and a government formation stage with parties that vary in the degree to which they are office-motivated and policy-motivated (see S7 Appendix for an ABM-ODD protocol of the model [22,23]). Parties neither know the electoral consequences of a policy shift nor do they know their rivals’ policy and office interests or their policy ideal points [7,9,24]. Parties do observe a party’s expressed policy position which need not be its ideal position. Since parties’ payoffs also depend on other parties’ actions via government formation and government policy-making, the maximization of utility becomes a very hard problem to solve. To cope, parties rely on heuristics to change their policy positions which affects their odds to become a member of government and affect government policy themselves.

The model is dynamic and proceeds in three steps. First, parties apply decision rules to change their expressed policy positions in a two-dimensional policy space. Note that party ideal points do not change. Second, elections take place and parties learn their electoral support. We contend that voters always vote for the party closest to their ideal points, that voters’ ideal points are two-dimensional, fixed over time and normally distributed, and that the electoral system is perfectly proportional. Third, parties use their electoral support to form a government which also sets the government policy position. Then, the model returns to parties shifting their positions and so on. We now detail parties’ utility functions, before explaining how parties form governments. Finally, we discuss decision rules parties apply to change their policy positions.

### Parties’ utility functions

Parties and governments take policy positions on two policy dimensions, x and y. Most party systems can be captured well by two dimensions [25]. Let *x*^*k*^ and *y*^*k*^ denote party *k*’s (time-independent) ideal point on these dimensions, respectively. Moreover, let $x_{t}^{g}$ and $y_{t}^{g}$ be the government’s policy position on these dimensions at time *t*. We assume that parties’ utility decreases in the squared distance between their ideal policy position and the government position. Other distance measures are reasonable choices as well [26]. Following Laver and Sergenti [4], however, we opt for theoretical continuity and comparability with the vast literature on party competition that uses squared distances. We set the maximal utility parties can gain from policy to 1 and the minimal utility to 0, then, party *k*’s policy payoff at time t is:

$$p_{t}^{k}=1-{\left(x^{k}-x_{t}^{g}\right)}^{2}-{\left(y^{k}-y_{t}^{g}\right)}^{2}.$$

If a party manages to set government policy at its ideal point, it does not incur policy loss (i.e., $p_{t}^{k}=1$). At the same time, the further away government policy is from a party’s ideal point–in either dimension–the larger its policy loss and the smaller $p_{t}^{k}$. Note that in calculating utility parties compare government policy to their ideal points *x*^*k*^ and *y*^*k*^ and not to their expressed positions denoted by $x_{t}^{k}$ and $y_{t}^{k}$. The latter are relevant for voters, and government formation (see below).

Once elections have taken place, parties learn their electoral support, $e_{t}^{k}$, which is also their seat count in parliament since we assume that the electoral system is perfectly proportional. Once a government is formed (see below), we know a party’s relative contribution of parliamentary support to the government. Formally,

$$s_{t}^{k}=\frac{e_{t}^{k}}{\sum_{1}^{J}e_{t}^{j}},$$

where j, for *j* ∈ {1,…,*J*}, are all *k* that are member of the current government in round *t*. Note that $s_{t}^{k}=0$ if *k* is not part of the current government, $s_{t}^{k}=1$ if *k* forms a single-party government, and $s_{t}^{k}\in (0,1)$ if *k* is member of a coalition government. We assume that Gamson’s law holds [27–29], and hence $s_{t}^{k}$ is also a measure of how many seats at the cabinet table party *k* controls.

We are now ready to define party *k*’s utility function which, in line with the literature on party motivations [8], is the sum of office pay-off (*s*) and policy pay-off (*p*) weighted by the party’s preference for policy or office payoffs (α).

$$U_{t}^{k}=\alpha \times p_{t}^{k}+(1-\alpha ){\times s}_{t}^{k}$$

The factor *α*, for *α* ∈ [0,1], by which these components are weighted, describes whether a party is policy-motivated or office-motivated. If *α* = 0, policy yields no payoffs and the party is perfectly office-motivated and tries to maximize its seat share at the cabinet table only. If *α* = 1, however, the party does not care about being a government member *per se* but rather about whether government policy is close to its ideal position. Finally, if *α* ∈ (0,1), parties value both policy and office, yet, policy becomes more important as *α* increases.

Note that parties do not obtain utility for choosing a policy platform close to their ideal points, instead, parties can only affect their utility by affecting the composition of government, the government’s policy position, or both. We now turn to how parties form governments.

### Government formation

We suggest a procedure for government formation that takes parties’ limited ability to deal with the high complexity of party competition into account. To our knowledge, there is no government formation procedure with boundedly rational actors yet [13]. In recent years, scholars have revealed on several occasions that the predictions of the canonical rational bargaining models [30,31] helped scholars greatly to explain several bargaining outcomes, however, their explanatory power is limited and some of their predictions fail to find empirical support [29,32,33]. More recently, new routes to modeling government formation have been chosen that deemphasize the role of formateurs [13,32,34]. The government formation procedure we suggest follows this type of models by delegating the task to aggregate parties’ preferences to a non-partisan head of state. We now describe to what extent parties are not able to cope with the complexity of government formation, and how they can form a government nevertheless.

Parties have a hard time to predict government formation outcomes because they neither know rival parties’ true policy ideals nor their appetite for office or policy, and hence cannot immediately predict whether a rival party likes a particular government or not. Just as we assume that parties are not able to know what moves to make in the policy space and thus use heuristics as cognitive shortcuts [7,9], we, for above reasons, maintain that they are not able to know what choices to make when forming coalition governments either and, hence, rely on heuristics to make these decisions as well. We argue that the heuristic they use to decide on a suggested government is to support any government that they prefer over the caretaker government that is installed if no other government forms. Effectively, this decision rule is a satisficing heuristic with caretaker government utility as aspiration level [35].

Besides being the logical extension of the heuristic approach to government formation [13], our government formation procedure–which we describe in detail below–has two more desirable characteristics. First, it is based on the “two constitutional constraints that exist in *all* parliamentary systems: (1) an incumbent government always exists, and (2) all governments, in addition to having the support of their member parties, must enjoy majority legislative support” [13,32]. Second, unlike many rational choice models of government formation [19] it gives rise to all types of governments including coalition governments, minority governments, oversized governments, and caretaker governments. Nevertheless, it ensures that single-party majority governments are formed whenever a party obtains a majority of seats in parliament.

In a nutshell, parties evaluate all potential governments that can form after an election, communicate their preferences over governments to the non-partisan head of state who suggests a candidate government. Unable to deal with the complex interactions of many parties’ preferences, parties simply compare the candidate government to the lurking caretaker government, and accept or reject it until either a government is installed or the current government is re-installed as caretaker government.

When evaluating governments, parties care, due to their utility functions, about two aspects: the government’s policy position and their own party’s share of seats at the cabinet table. Since we assume that Gamson’s law holds, parties’ share of seats at the cabinet table is given by $s_{t}^{k}$. Moreover, we contend that a government’s policy position is simply the average expressed policy position of all cabinet parties weighted by $s_{t}^{j}$: Specific examples of real-world bargaining may significantly deviate from the weighted cabinet mean position or Gamson’s law. Nevertheless, both are on average good approximations of government formation outcomes [29,36], and particularly so in uncertain circumstances [34,37].

$$x_{t}^{g}=\frac{1}{J}\sum_{1}^{J}s_{t}^{j}x_{t}^{j}$$

and

$$y_{t}^{g}=\frac{1}{J}\sum_{1}^{J}s_{t}^{j}y_{t}^{j}.$$

Please note that once elections have taken place, every potential government’s distribution of seats at the cabinet table as well as its policy position are public knowledge and parties have all information at hand to compute their own utility if each potential government formed. However, two crucial elements of rival parties’ utility functions are unknown to parties, i.e. the extent to which they favor policy over office (i.e., *α*) and their policy preferences (i.e., *x*^*k*^ and *y*^*k*^). Hence, deriving rivals’ preferences over governments from this information is a highly challenging task which, similar to predicting the results of policy shifts, parties are unable to cope with [9]. We, therefore, suggest that parties report their preferences over governts sincerely to a non-partisan head of state and compare any suggested government to the current government staying in office (caretaker government). This gives rise to the following government formation procedure:

1. Parties compute the utility they would obtain from all potential governments–including governments they do not participate in, oversized and minority governments.
2. Parties rank all of these governments according to their utility. They report their ranking to a non-partisan head of state who will suggest a government to the parties in step four.
3. The head of state weighs the preferences revealed to her by parties’ parliamentary seat shares. This implies that large parties’ preferences are more influential.
4. The head of state suggests the highest ranked government to parties.This way, parties evade voting cycles [31].
5. Each party compares the utility it receives from the suggested government to the scenario in which the current government remains in office as caretaker government. This is a satisficing heuristic with caretaker government utility as aspiration level. Since caretaker governments cannot pass legislation as easily as ordinary governments, the utility parties receive from caretaker governments are discounted by a factor which is a model parameter we vary. If the suggested government yields at least as much utility as the lurking caretaker government, a party signals its support for the candidate government to the head of state.
6. Having learned parties’ support for the candidate government, the head of state evaluates whether the candidate government is supported by (1) all members of the candidate government and (2) a majority in parliament [13]. If either of these conditions is not met, the head of state repeats step 4 (i.e., suggesting the highest ranked government to parties), yet, she erases the just suggested candidate government from her list. This procedure continues until either a candidate government is supported by all of its members and a majority in parliament, or all candidate governments are rejected and the caretaker government is installed [32].

We now turn to the decision rules parties use to adjust their policy positions.

### Party decision rules

Each decision rule represents a heuristic that is likely to help party leaders compete in the policy space given a particular incentive structure (for example, electoral system or intra-party politics; [4,7]). When Laver and co-authors [4–7] studied parties’ decision rules, they measured party success as party vote share. We introduce policy and office preferences to the model and can, hence, compare the performance of rules developed for a vote-seeking world with rather government-centered rules. In particular, we adopt three of the decision rules studied by Laver and his co-authors (i.e., Aggregator, Hunter and Sticker), yet, we also suggest two new rules which use the government as a point of orientation (Governator and Satisficing Governator). We limit the maximal distance a party can move each round to .12 units [4] in the policy space. Hence, it would take about 12 moves to travel the maximum ideological distance on a straight line.

**Aggregator** [4]: Identify the mean coordinate on each dimension of the ideal points of your current party supporters; move in this direction unless this causes you to overshoot, in which case move to the mean of supporter ideal point.

The real-word party type that is most similar to Aggregators is a highly decentralized party that gives a lot of policy-making authority to its members and supporters. For instance, many Green parties have strong intra-party mechanisms that tie party policy to membership decisions [4,38,39].

**Hunter** [4]: If the previous move led to fewer votes, reverse direction and move on a heading randomly selected within the half-space now being faced. Otherwise, move in the same directions as last round.

In currents politics, Hunters are most likely vote-seeking parties that do not have too strong ideological roots. They can easily adjust their party policy to election results because being successful at elections mutes intra-party challengers. Laver and Sergenti [4] argue that many centrist catch-all parties resemble Hunters. In fact, Budge [9] describes party policy shifts as zigzag movements: parties move slightly to the left, and at the next election slightly to the right. This can be seen as typical Hunter behavior.

**Sticker** [4]: Stay put.

Stickers never change their ideological position, irrespective of electoral incentives. They are ideologically highly cohesive and usually very small niche parties. Examples are religious minority parties (e.g., in the Netherlands or Israel).

**Governator:** Move toward the government position unless this causes you to overshoot, in which case move to the government position.

The crucial innovation of the Governator rule is that it is focused on the government’s policy position. The underlying idea is that a shift toward the government’s position is very likely to make a party member of the next government coalition. This notion is obviously very appealing to office-seeking party leaders. At the same time, however, policy-seeking party leaders may use this decision rule to move their party to a policy position that allows for government participation that results in a better government policy position.

**Satisficing Governator:** If member of the current government, stay put. Otherwise, move toward the government position unless this causes you to overshoot, in which case move to the government position.

While the Governator keeps on moving until a party reaches the government’s position, the Satisficing Governator rule stays puts as soon as the party enters the government. Acting in an environment of major uncertainty, satisficing is an attractive decision feature [4,40]. The Satisficing Governator, thus, does “not fix what is not broken” as long the party is in government. Yet, the party orients toward the government position when excluded from government.

## Two measures of representation

Our ultimate goal is to learn about different rules’ and model parameters’ implications for political representation of voters. When discussing voter representation, we acknowledge that there are different ways to evaluate representation [18], and party systems that perform well in terms of how the legislature represents the electorate, may perform less well in terms of how the government represents the electorate [41]. Before discussing our operationalizations, however, we introduce a measurement of party policy extremism that is crucial for both explaining the dynamics we observe and for verifying the data estimation procedure.

### Party policy extremism (eccentricity)

As a measure of how policy extreme a given party system *p* is, we compute mean party system eccentricity (*E*_*p*_). We define it as parties’ average distance from the mean voter policy position. Formally,

$$E_{p}=\frac{1}{K}\sum_{k=1}^{K}\sqrt{{\left(x_{t}^{k}-{\overset{-}{x}}^{v}\right)}^{2}+{\left(y_{t}^{k}-{\overset{-}{y}}^{v}\right)}^{2}}$$

where ${\overset{-}{x}}^{v}$ and ${\overset{-}{y}}^{v}$ are the mean positions of voters on the policy dimensions, respectively. The higher E_p_ the more aggregate distance between parties and the mean voter.

### Party system misery

Following Laver and Sergenti [4], we understand a party system to represent voters well if every voter has a party that takes a position close to the voter’s ideal position. In particular, the smaller the squared distance between voters and their most proximate party, on average, the better political representation. In terms of Golder and Stramski [18], this is a many-to-many understanding of political representation.

Formally, we measure system misery, *S*_*p*_, in a party system, *p*, as

$$S_{p}=\frac{\sum_{v}d(i_{v},j_{vp})²}{n}$$

where *d*(*i*_*v*_,*j*_*vp*_) is the Euclidean distance between voter i’s ideal position and the policy position of the party closest to her in *p*, *j*_*vp*_, and *n* is the number of voters. The smaller this figure, the better a party system represents voters.

### Government misery

Even if party system misery is low, government policy may, nevertheless, be very unrepresentative of the electorate [41]. To measure congruence between voters and the government policy (a many-to-one relationship), we marginally adjust Golder and Stramski’s [18]) measure of relative citizen congruence. It ranges from 0 to 1 and captures “the average [squared] distance of a citizen from the citizens’ most preferred position relative to the average distance of a citizen from the government”. Formally we measure government misery *G* in party system, *p*, as

$$G_{p}=1-\frac{\sum_{v}d{\left(i_{v},m\right)}^{2}}{\sum_{v}d{\left(i_{v},g_{t}\right)}^{2}}$$

where ***m*** is the median citizens’ positions (using the L1 median concept to find the median in multiple dimensions) and ***g***_***t***_ is the government policy position at time *t*. The smaller ***G***_***p***_ the better political representation by the government.

## Linking model input to model output

We seek to understand how model parameters and decision rules affect the measures of representation. We face two challenges in obtaining meaningful results: Ensuring that the model output we report is representative for the corresponding model input, and describing how model output varies systematically with model input. We discuss these briefly and refer to S1 Appendix for more detail.

To ensure model output represents model input correctly, we follow Laver and Sergenti [4] and treat the agent-based model as a Markov chain whose steady-state distribution of our measures (eccentricity, party system misery, government misery) are our quantities of interest. Since these steady-state distributions are virtually always too large to be mapped out, we compute ensemble averages of 500 burnt-in Markov chains using identical model inputs and different random numbers. In S1 Appendix we show that the ensemble averages are based on steady-state data, and do not change too much when more than 500 Markov chains are used to compute ensemble averages. In total, we argue that our decisions are very close to the sweet spot between computational effort and high quality data.

To evaluate how model output varies with model input, we conduct the equivalent of an experiment by setting model input exogenously. We draw model parameters (except for rules) randomly from their parameter space. Hence, the variability in ensemble averages we obtain can be decomposed into three types of variability: variability due to ensemble average error, variability systematically caused by model input (e.g., more eccentricity with vote-seeking rules), variability due to random factors (e.g., the random initiation of party systems). The checks discussed in S1 Appendix suggest that ensemble average error is small. Thus, any substantive changes in model output result from model input or are due to random idiosyncrasies of a given model. Multivariate regression techniques are designed to uncover the marginal effects of individual model parameters on model output in such circumstances. They are, however, sensitive to the functional form of variables included in the model. To nevertheless recover the “true” data generating process, we data mine using the polywog procedure [42,43]. Relying on k-fold cross-validation, this algorithm finds the regression equation including model parameters’ polynomials, (their) interactions and dummy variables for extreme parameter values, that links model input to model output best. We then use this regression equation to judge how individual model parameters shape our quantities of interest. Hence the predictions we make are expected means (i.e., regular linear regression predictions) of ensemble averages, or to put it differently: expected means of means. Given that our data is experimental data, we are confident that this procedure allows us to evaluate the relationship between model input and model output precisely. We find that the predicted model output is very close to the actual model output. A slight exemption is the Hunter rule. Its behavior is subject to more randomness than other rules, and consequently over all model fit and standard errors are larger. For instance, for the single rule simulation case, the regression modeling Aggregators’ eccentricity explains 99.49% of variance, whereas the corresponding Hunter regression explains only 80.61% of variance. Nevertheless, even the results for Hunters are robust and clearly linked to model input. S1 Appendix clarifies this procedure in more detail.

## Results

**Fig 1:**
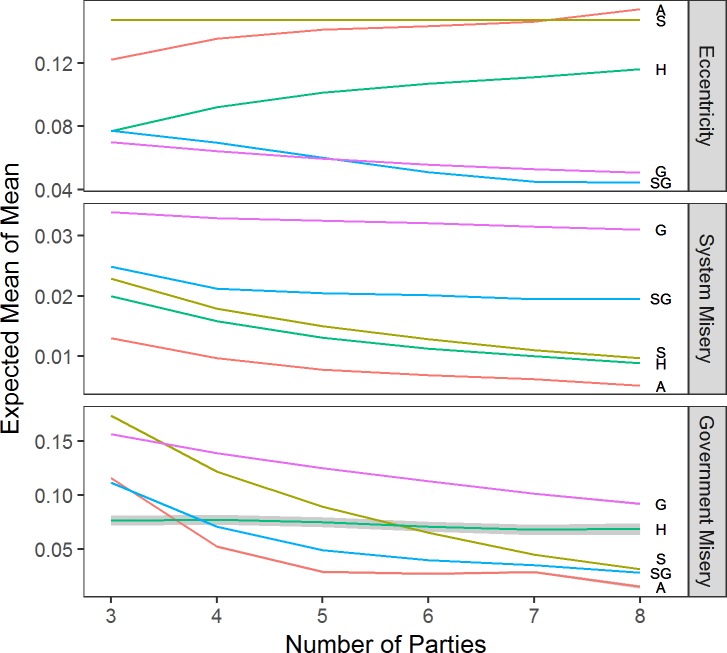
The effect of number of parties. *Note*: Based on corresponding data mined OLS regressions with α at .5, discount factor at .5, and ideal point dispersion factor at 1. Grey shaded areas are 95% confidence intervals. A = Aggregator, S = Sticker, H = Hunter, G = Governator, SG = Satisficing Governator.

We first turn to the results of the one-rule models, i.e., all parties in the party system use the same decision rule to shift their policy positions. Fig 1 presents the simulation results for different rules. We place these in the same plot to facilitate comparisons between models and rules.

### Party-system size

The first important insight is that as the number of parties increases (see Fig 1, top panel), both types of government-oriented rules (Governator and Satisficing Governator) take more centrist positions (less eccentric), while vote-oriented rules (Hunter and Aggregator) are more eccentric. What is the reason for this? Laver and Sergenti [4] show that as the number of parties increases the center becomes overcrowded, and hence, vote-oriented parties face incentives to move away from the center. Office-oriented rules, by contrast, move toward the government position which is typically rather centrist, especially the more parties are competing.

Ordinary voter do not care about party eccentricity. They care about whether there is a party that expresses their views (system misery) and whether government policy is in line with her ideals (government misery). Turning to system misery first (center panel in Fig 1), we find that no matter what rule parties use, on average, system misery decreases as more parties compete. This means that voters have a more proximate party to vote for as the number of parties increases. Also, a randomly added party (i.e., an increase in the number of Stickers) decreases party system misery more than the addition of a government-oriented party (either type of Governator). By contrast, vote-oriented rules perform roughly as well as a randomly positioned party. Put differently: Another party on the voters’ menu improves their representation in parliament, on average. However, vote-oriented rules (Aggregator and Hunters) reduce system misery more because these will position at niches that are not well represented yet.

The bottom panel of Fig 1 shows that also government misery decreases with more parties. Stickers perform badly when there are only few parties in a party system. As the number of parties increases, government misery increases rapidly (by more than factor 2). Not so with Governators. They perform badly with few parties, and improve their performance with more parties but at a much lower rate than other rules. This is because once a government is stable for several iterations, all parties converge to its position. The government that forms once all parties have converged to the same policy position is unchallenged, however, its policy position need not be centrist. In fact, where parties end up is mainly determined by where they are initiated. As a result, with eight parties in a party system, Governators produce roughly three times the government misery that Stickers produce.

Satisficing Governators, on the other hand, perform, on average, better than Stickers. The differences between the two types of Governators are rooted in the early iteration of a simulation run. Satisficing Governators in office do not converge to the (centrist) government position they do so only when in opposition. With opposition Satisficing Governators shifting to the center of the party system, the governing parties profit by picking votes on the flanks. As the latter parties grow stronger, they can turn to the now smaller, and more centrist opposition parties to form a government. This makes the government’s position more centrist. We now move to discussing the effect of other simulation parameters on system misery and government misery.

### Degree of policy-seeking and parties’ ideological distance

**Fig 2:**
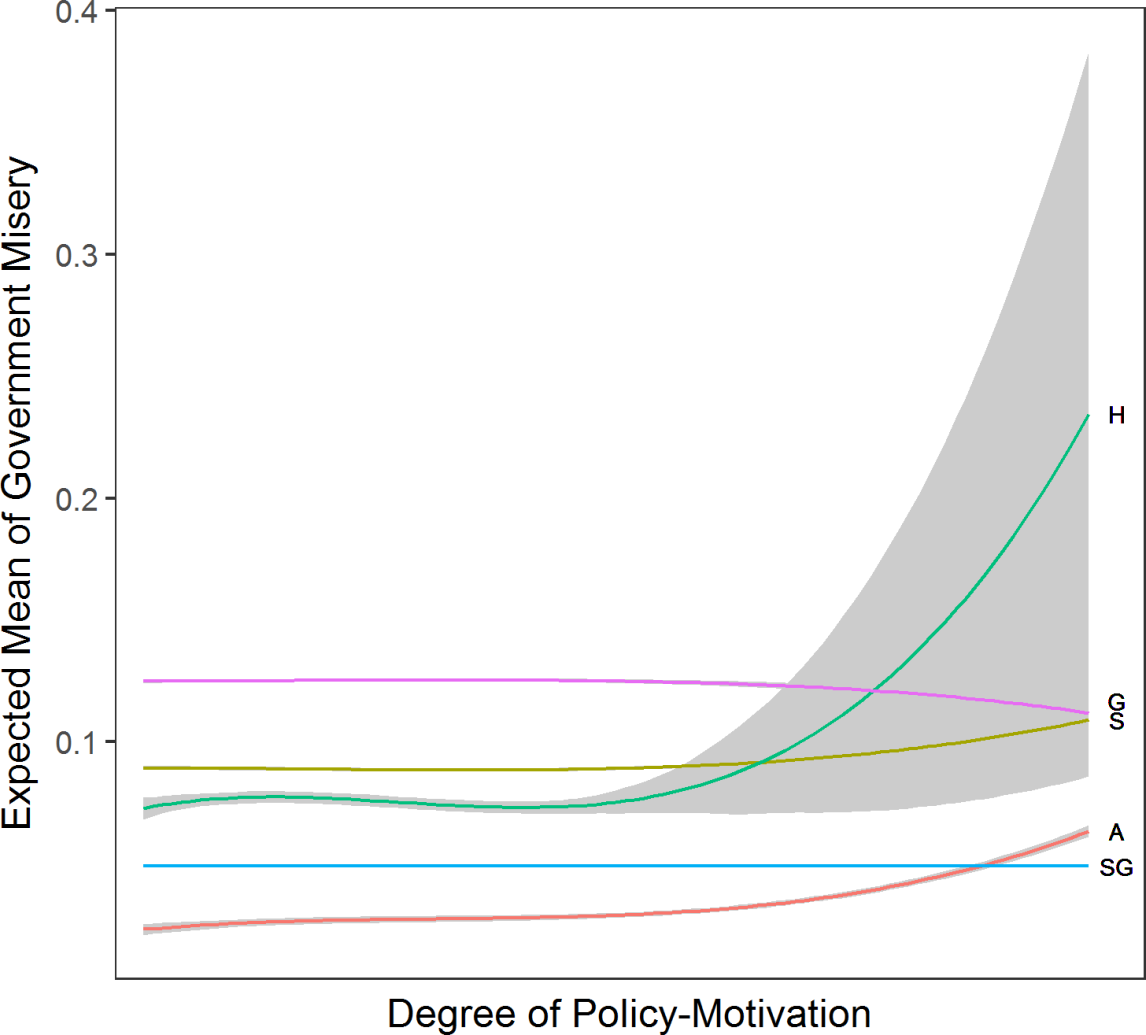
Effect of degree of policy-motivation. *Note*: Based on corresponding data mined OLS regressions with 5 parties, standard ideal point variance, and discount factor at .5. Grey shaded areas are 95% confidence intervals. A = Aggregator, S = Sticker, H = Hunter, G = Governator, SG = Satisficing Governator. The x-axis shows the degree of policy-seeking of a party, where 0 is fully office-motivated and 1 is fully policy-motivated.

We first analyze the effect of the degree of parties’ policy-seeking on government misery (see Fig 2). We find that the effect of policy-seeking is rule-specific: for parties who value policy more than office (α > .5), government misery increases when parties apply Aggregator, Sticker or Hunters rules. Note also that with respect to government misery, Satisficing Governators and Aggregators produce less misery than Stickers, Hunters and Governators for all degrees of policy-seeking.

### Dispersion of party ideal positions

**Fig 3:**
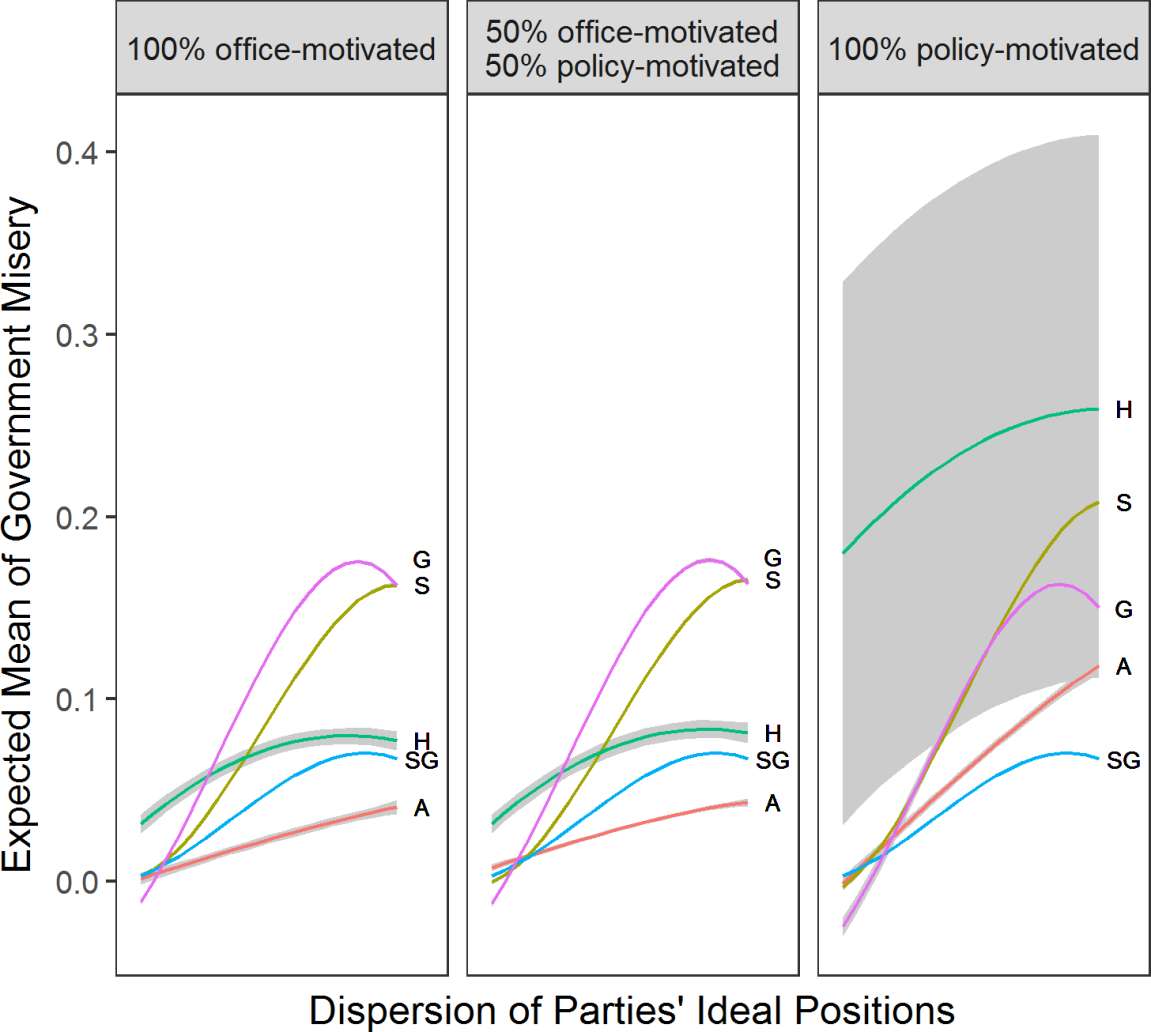
Effect of dispersion in parties’ ideal positions. *Note*: Based on corresponding data mined OLS regressions with 5 parties and discount factor at .5. Grey shaded areas are 95% confidence intervals. Columns represent different levels of policy-motivation. A = Aggregator, S = Sticker, H = Hunter, G = Governator, SG = Satisficing Governator.

Fig 3 shows that as the dispersion factor in parties’ ideal positions increases (i.e., parties’ ideal points become more polarized, x-axis), government misery increases (y-axis). This is hardly surprising as no ideal point dispersion implies that all parties’ ideal points are identical and very close to the center of the voter distribution. Furthermore, the strength of the effect hinges on parties’ level of policy-motivation (columns in Fig 3) and the decision rule. Hunters and Aggregators cause much higher government misery at high levels of ideal point dispersion if they are fully policy-motivated.

Some of the differences between rules stem from the different types of governments they form. We find that the office-oriented rules produce on average more ideologically cohesive governments, and Governators in particular are much more likely to join oversized majority cabinets (see Fig A in S3 Appendix). This is despite the fact that the procedure that parties follow to form governments does *not* vary across decision rules.

When comparing effect sizes, the number of parties in a party system stands out as the most important predictor. The other parameters matter significantly less (see for example effect of discounting in S2 Appendix), except for some rather extreme combinations of parameters (e.g., completely office-motivated parties and changing discounting of caretaker governments).

Overall, irrespective of the rule used more parties are better for political representation both by the government and the party system. Yet, not all rules perform equally well. Clearly, Aggregators perform best at both types of representation (except for some extreme cases). Finally, as parties become more policy-seeking, government misery increases with Hunters, Aggregators, and Stickers. We now turn to the evolutionary models to learn how rules perform when being exposed to other rules as well.

## Evolutionary simulation

In the evolutionary models, multiple rules can exist in the same party system. Moreover, parties are able to change the rules they use to choose policy positions. We first describe when and how parties change rules before describing to what extent parties actually change rules, and if certain rules dominate party systems. We then turn to evaluating how political representation is affected by different rules and model parameters in the evolutionary context.

### Experimental design

In the evolutionary model we introduce two new model parameters: memory (*m*) and aspiration (*a*). Memory dictates the number of rounds (2 through 10) a party can look back to. Aspiration, denoted *a*, indicates a threshold level of utility, a new model parameter which is randomly assigned and takes the values .25, .5, .75, or .9, which makes parties choose a new decision rule if it is not met. At the end of each iteration, parties follow the following procedure:

1. If party *k* used the same rule in the last *m* rounds, compute *k*’s mean utility of the last *m* rounds, $U_{m}^{k}$.
2. If $U_{m}^{k}$ < *a*, choose a new decision rule randomly.

If some rules gain significantly less utility than other rules, they will gradually disappear from the population of rules that parties apply. At the same time, they are unlikely to disappear altogether because a new decision rule is selected with equal probability. S4 Appendix summarizes the simulation set-up in detail.

### Analysis of rule changes

Now that we allow for evolution, parties can change the rules they use. This raises a number of questions which we will answer before discussing the representation results of the simulations.

**Fig 4:**
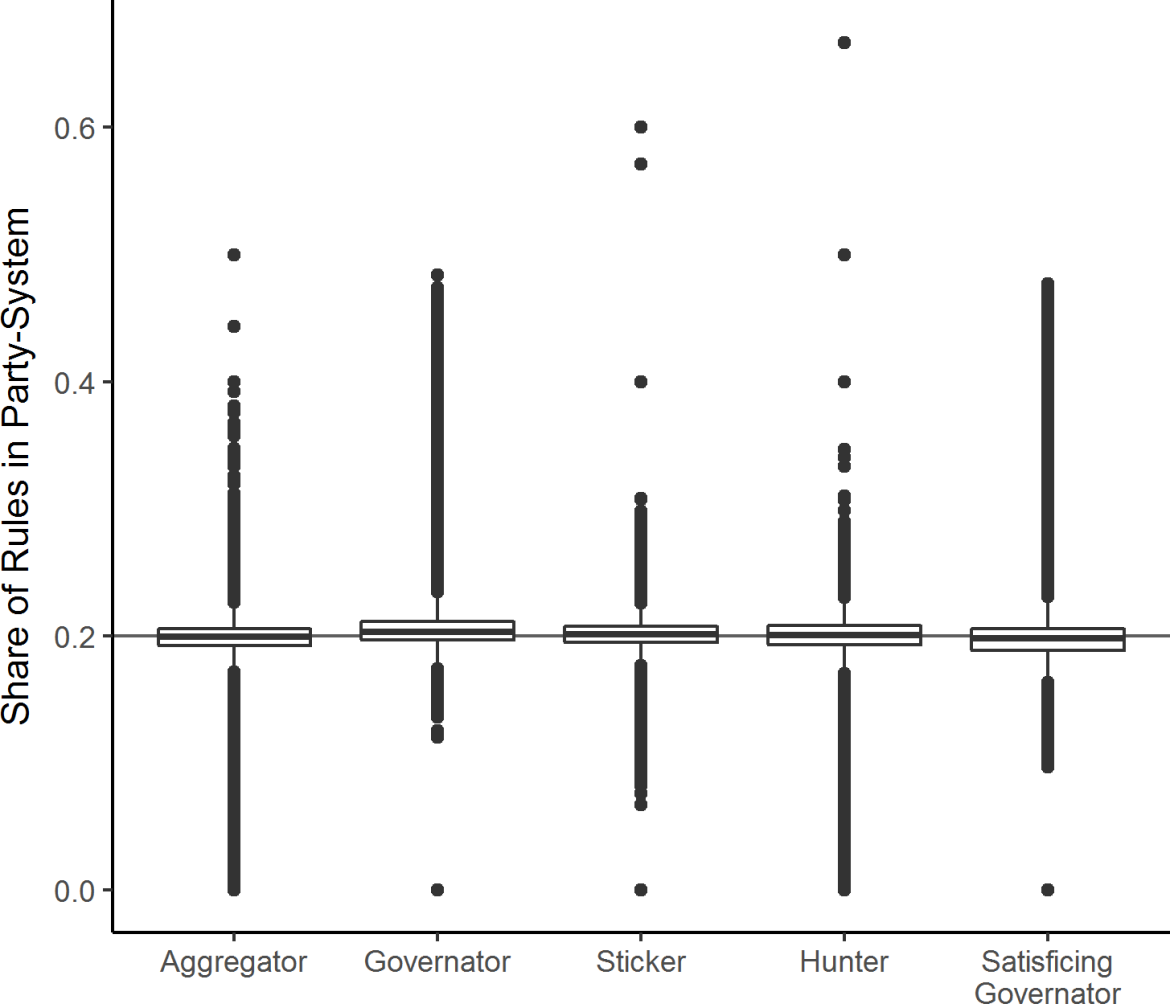
Boxplot of rule shares in simulated party systems.

Does one rule dominate the simulated party systems? No, as Fig 4 shows, each rule’s median share over all model runs is almost exactly 20%. Only Governator occurs more often and Satisficing Governator less often, but these margins are tiny. Moreover, at most 50% of parties apply the same rule in a given party system.

Do some rules frequently appear together? Yes, both types of Governators (r = .66), and Hunters and Aggregators (r = .76) co-occur (Table A in S5 Appendix). At the same time, party systems with many vote-oriented rules tend to have few office-oriented parties (e.g. the correlation between Governator and Aggregator is -.83). There does seem to be co-evolution of similar rules, even though they have quite different outcomes in terms of representation.

How often does rule change occur and which factors stimulate or depress rule change (for full discussion see S6 Appendix)? By design, the memory parameter controls how often parties can change rules within the observed period. If parties can change rules every other iteration (*m = 2*), the average share of rule changes per round has its theoretical maximum at .5. Put differently, if every party changes rules every *m* rounds, we would expect a share of 1/*m* parties to change rules each round. We find that fully office-motivated parties indeed change according as much as theoretical maximum allows (Fig A in S6 Appendix). The more policy-motivated parties are, the less frequent rule change becomes. Fully policy-motivated parties rarely change rules (Fig B in S6 Appendix). As for the other new parameter in the evolutionary model–aspiration–parties with the lowest level of aspiration (.25) change rarely, especially the more policy-motivated they are. For higher levels of aspiration rule change increase dramatically, except if parties are fully policy-motivated (Fig D in S6 Appendix). Finally, if parties care more about office than about policy the number of parties also drives up the frequency of rule change (Fig C in S6 Appendix).

In total, these results suggest that our model behaves in meaningful ways, e.g., that policy-motivated parties change rules less often. This is reasonable because government policy is a public good that all parties have access to even when they are not in government. Put differently, a party’s policy payoff may be small but it is virtually never non-existent. Office utility, however, is derived from private goods (government seats) from which a party can easily be excluded with severe consequences for its utility.

### Analysis of representation variables

What are the effects of the rules on eccentricity and system misery and government misery? Are they the same as in the non-evolution simulation? To evaluate this we again follow the polywog procedure described earlier, but this time we add rule shares as independent variables. The Sticker rule share is left out and functions as reference category. The model parameters and rule shares explain most of the variation in eccentricity (93.2%), system misery (96.1%) and government misery (83.2%).

**Fig 5:**
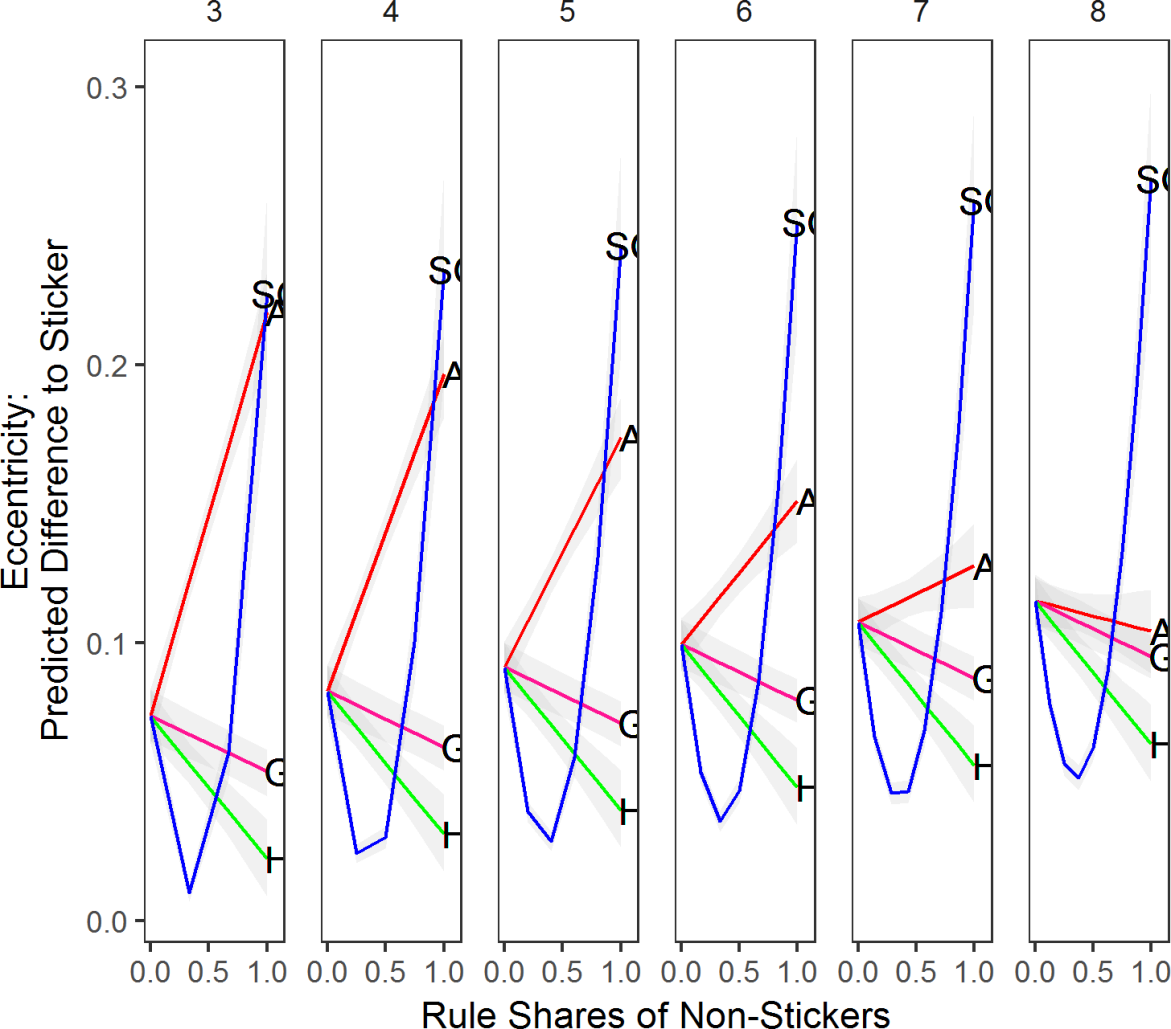
Predicted eccentricity for rule shares compared to sticker by number of parties. *Note*: Based on corresponding data mined OLS regressions with all parameters at mean value, and other rule shares kept at 0. Grey shaded areas are 95% confidence intervals. Columns represent the number of parties in the party system. A = Aggregator, S = Sticker, H = Hunter, G = Governator, SG = Satisficing Governator.

We start with analyzing eccentricity. In Fig 5 we plot the effect of increasing the number of rules in a party system by one party at a time, holding all other rule shares at 0. The 0 rule share point in the graph is the point where all rules are Stickers. There are a few differences in terms of eccentricity between the evolutionary and non-evolutionary simulations. First, the Sticker rule is not as eccentric as before, as eccentric Stickers simply change rule due to low utility. Second, the Aggregator rule takes the most eccentric positions, but this becomes less so the more parties in the system. Third, at first more Satisficing Governators lower eccentricity, but when almost all parties in the system use the Satisficing Governator rule, eccentricity becomes very high. What happens is that Satisficing Governators get stuck somewhere in the party system, and when there is no vote-seeking rule (e.g. Hunter or Aggregator) in the system, there is essentially no mechanism to draw them out of their eccentric position. Fourth, Governator rules do not suffer from this dynamic, and are relatively uneccentric.

What are the effects of the model parameters? More parties lead to slightly more eccentricity. More ideal point dispersion changes the effects of rule shares reported above. In particular, when there is more ideal point dispersion Satisficing Governators become more eccentric compared to Stickers, and Governators become less eccentric. More policy-motivation leads on average to more eccentric parties, and particularly Hunters become much more eccentric, and Governators less eccentric. Aspiration, on average, reduces eccentricity for the office-oriented rules. The vote-oriented rules, and in particular Aggregator becomes a lot more eccentric. Discounting and memory have almost no effect.

**Fig 6:**
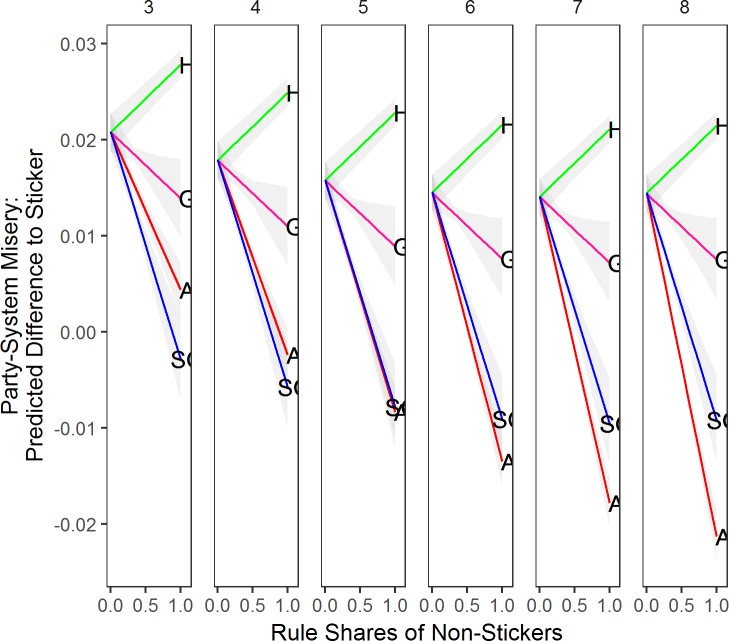
Predicted party system misery for rule shares compared to sticker by number of parties. *Note*: Based on corresponding data mined OLS regressions with all parameters at mean value, and other rule shares kept at 0. Grey shaded areas are 95% confidence intervals. Columns represent the number of parties in the party system. A = Aggregator, S = Sticker, H = Hunter, G = Governator, SG = Satisficing Governator.

Now we move to the analysis of party system misery (see Fig 6). Due to its eccentricity Aggregators provide the best party system misery in party systems with more than 5 parties. Interestingly, the Satisficing Governator does not achieve this despite its eccentricity. The rationale for this is that Satisficing Governators can get stuck in an area of the two-dimensional space where a government is initially formed. Governators provide slightly lower sytem misery than Stickers, but Hunters underperform compared to Stickers (high misery). Besides individual rule effects, Fig 6 also demonstrates a strong effect of the number of parties. The more parties compete, the less party system misery we observe.

Do other model parameters explain party system misery? Ideal point dispersion affects only the effect of Hunters on party system misery. The more dispersed ideal points are, the more party system misery Hunters produce. Policy-motivation increases party system misery a little, as parties become more eccentric. Memory, aspiration and discounting have negligible effects. While the results are not exactly like the single-rule simulations’ results, they are highly similar.

**Fig 7:**
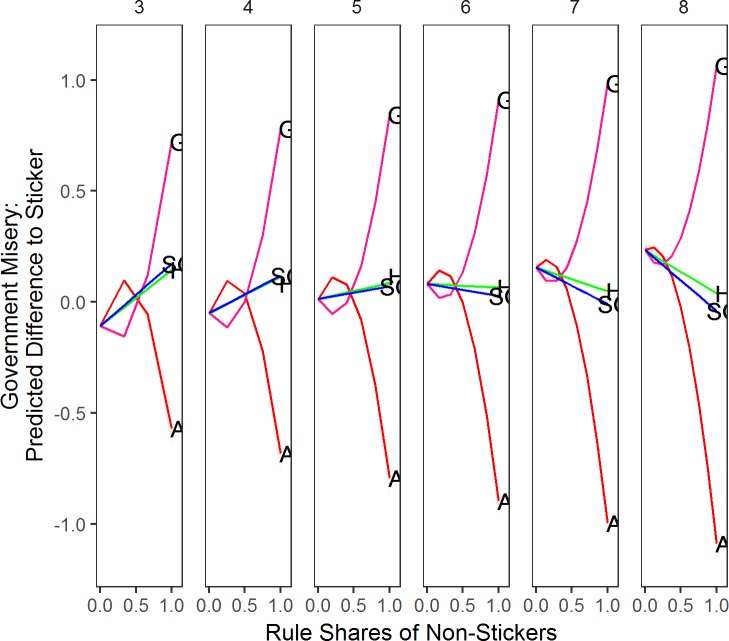
Predicted government misery for rule shares compared to sticker by number of parties. *Note*: Based on corresponding data mined OLS regressions with all parameters at mean value, and other rule shares kept at 0. Grey shaded areas are 95% confidence intervals. Columns represent the number of parties in the party system. A = Aggregator, S = Sticker, H = Hunter, G = Governator, SG = Satisficing Governator.

Fig 7 presents the results for our analysis of government misery. If we add one Aggregator to an all-Sticker system the effect is negligible in 3- or 4-party systems. The more Aggregators we add the more government misery gets progressively reduced. In general, Aggregators can form governments that represent citizens well as soon as they can collaborate with a sufficient number of Aggregators. Adding one or two Governators to a system somewhat reduces misery, but adding more Governators increasing misery substantially because all parties converge to the relatively unrepresentative government position. Hunters’ and Satisficing Governators’ effect on government misery hinge also on the number of parties in the party-system. With three to five parties, they lead to more government misery as their number in the party systems increases. When there are six or more parties, more Hunters and Satisficing Governators results in less government misery.

Also other factors influence government misery. More policy-seeking leads to more government misery for all rules except for Stickers. In very polarized environments government misery is higher on average. As parties’ aspiration level increases, Aggregators and Hunters produce less government misery.

In conclusion, we find that more parties are better for representation, yet we also observe that the rate at which they reduce misery hinges on the decision rules they apply. In the evolutionary simulation, Aggregator parties still outperform other parties with respect to party system misery and government misery. Finally, the degree of policy motivation of parties increases government misery. We now contrast results from both simulations, and derive hypotheses from them.

## Conclusion

Simulation Results: What is best for representation? Table 1 summarizes our main findings. We rank-ordered parties on the two different misery variables. This rank-ordering is based on 5-party systems. In the simulations with a single rule (see Fig 1) we declare Aggregator the winner. It outperforms all other rules in terms of government misery and party system misery. We declare Hunter the runner-up, and Governator is a rule better to avoid. What about the evolutionary simulations? There again we give the gold medal to the Aggregator in the competition for low party system misery (see Fig 6) and low government misery (see Fig 7).

**Table 1:** Ranking of rules in each simulation.

	Simulations with one rule		Simulations with evolution	
	Party system misery	Government misery	Party system misery	Government misery
**Rank order of rules**				
Aggregator	1	1	1^*^	1^*^
Hunter	2	3	5	3
Sticker	3	4	4	2
Satisficing Gov	4	2	1^*^	3
Governator	5	5	3	5
**More** **policy-seeking**	Increases misery for SG and G; no effect otherwise	Rule-dependent effect	Increases misery	Increases misery
**More** **parties**	Decreases misery	Decreases misery	Decreases misery	Decreases misery
**Voter ideal point** **dispersion**	Increases misery for SG and G; no effect otherwise	Increases misery	Increases misery for H; no effect otherwise	Increases misery

Note: these rankings are based on Fig 1 (simulations with one rule) and Figs 6 and 7 (simulations with evolution). For each variable we have ranked the parties on the basis of their reduction of misery: 1 is best, 5 is worst. S = Sticker, SG = Satisficing Governator, H = Hunter.

* this ranking depends on the number of parties (see Figs 6 and 7).

The two simulations provide clear results regarding the effect of policy-seeking and party system size. Surprisingly, (pure) policy-seeking makes a government unrepresentative, and can have negative effects on party system misery. More parties generally lead to better representation, both in terms of what the party system and the government offer. However, we also find a robust pattern that some rule types reduce misery more than other rules. As for the other model parameters, we can conclude the following: the discount factor on caretaker governments is by-and-large unimportant (except when it is 0), the more diverse parties’ ideologies the more government misery. Memory and aspiration have negligible effects on the simulation output.

Hypotheses derived from results: From our results we derive three hypotheses that can be tested by empirical research:

H1: The Aggregator rule is best for democracy.

There is an extensive literature that explains variation in the quality of representation with system factors such as the electoral system [44,45,18,46,47]. However, party-level explanations are typically ignored [48,49]. Our paper demonstrates that how parties shift position has important consequences for representation. In fact, by focusing on the party-level we connect the literature that tracks individual parties’ policy changes [3] to the literature on the quality and satisfaction with democracy [50,51]. To our knowledge, such a link does not exist up until now, even though A problem here is that measuring the decision rules proposed in this article and by Laver [7] can be difficult. While some of the rules are easily observable (e.g., Sticker), others, however, are harder to discern because a party can shift towards the government position (both types of Governators) and repeat the policy shift of the previous election (Hunter) at the same time. Hence, real policy shifts cannot always be tied directly to one specific rule. To remedy this we need to map how parties formulate their policies We will also require information on whether parties are more concerned with changes in voter support and voter preferences (like Hunter or Aggregator) or with governing (like the Governator and Satisficing Governator). Promising research strategies are to analyze intra-party policy-making mechanisms as well as parties’ policy positions as expressed in speeches and press statements. Since these are available more frequently than party manifestos, it is easier to tease out effects of individual events (e.g., the changes in polling). Future research should, thus, engage more thoroughly in understanding how parties make decisions on policies, and to what extent rival parties [52], party members or party supporters [38,39], and the government serve as precedents for policy emulation [21,53].

Once a corresponding measurement is found, we can test Hypothesis 1. We can then also evaluate whether the rules presented here, that focus on formulating a policy position, carry over to other arenas of party politics. For instance, government parties may behave differently in policy-making depending on whether they use decision rules that focus on their party supporters (Aggregator) or on the general electorate (Hunter) [54].

H2: (Pure) Policy-seeking behavior increases government misery.

The pursuit of prestige and rents (office-seeking) is often seen as normatively undesirable, in contrast to political action in pursuit of policy goals. Our results back a different conclusion: government misery increases when parties have very strong policy motivations. One can test this assumption by using expert survey measurements of the degree to which parties are policy-seeking [55] with data on government misery. Laver and Sergenti [4] report that policy-seeking motivations also depress party system representativeness, a finding we did not replicate. This and our result suggest the importance for theoretical and empirical models of further distinguishing office-seeking and policy-seeking parties.

H3: More parties are better for party system and government representation. However, the addition of parties with some decision rules (Governator and Satisficing Governator) reduce misery more than parties with other decision rules.

As noted, the literature on representation primarily points to electoral system differences as an important explanatory factor [18,44–47]. Our simulations do not contrast single-member district electoral systems with proportional representation (PR) electoral systems. Yet, our results do imply that within multi-party systems additional variation in the congruence between voters and parties can be explained by the number of parties. More importantly, however, the results suggest that the number of parties interacts with the decision rules parties apply. Especially, adding more parties that apply the Hunter rule will not improve representation (by the party system and the government), while adding parties using other rules will. Our simulations demonstrate that the number of parties, and the rules they apply should be relevant predictors of party system misery and government misery.

Policy recommendations: In virtually all cases, Aggregators perform best in terms of voter representation by the legislature and the government. Furthermore, in Aggregator party systems there is no trade-off between party system misery and government misery. All governments consist of parties with rather different policy positions, but since voters are distributed equally over these parties, the government position is very centric. But how to get parties to act like Aggregators? We suggest that a system of primaries that gives voters the right to shape their parties’ policy positions or election manifestos may motivate parties to act like Aggregators. Research on intra-party democracy indicates that handing rank-and-file members control over leadership positions and policy decisions, makes parties’ respond to their supporters [38,39,56,57].

Limitations. There are several aspects which are beyond the scope of our paper. A first limitation is that voter preferences in our model are normally distributed. While and Laver and Sergenti[4]) show that such an assumption is warranted (see pp.50-55), they also show that other distributions–like multimodal ones—produce different results (pp. 104–105 and p.121). Future research should explore to what extent different decision rules perform well in split societies or societies in which voter preferences gradually shift or become more dispersed. A second limitation is that we study only one procedure of government formation. The literature on government formations suggests that the reason for termination of the outgoing government affects what new government is likely to form [58,59]. This is only one of many aspects that future research can look into to further understand the effect of government formation on policy outcomes and political representation. Finally, the set of parties decision rules we suggest is limited and many alternatives are feasible [5]. Future research should scrutinize how other aspects of party behavior (e.g., acceptance to perform badly for a number of elections to find a good policy position) can affect political representation.

## Supporting information

S1 Appendix Simulations single-rule case. (PDF)

S2 Appendix Effect of discounting the caretaker government. (PDF)

S3 Appendix Government characteristics in non-evolutionary model. (PDF)

S4 Appendix Burn-in length for evolutionary simulation. (PDF)

S5 Appendix Correlation between rule-occurrence in party systems. (PDF)

S6 Appendix Analysis of rule change. (PDF)

S7 Appendix ABM-ODD protocol. (PDF)

S8 Appendix Variability analysis. (PDF)
