# Supplementary material for: Governator vs. Hunter and Aggregator: A simulation of party competition with vote-seeking and office-seeking rules
Source: PLoS One. 2018 Feb 2;13(2):e0191649. doi: 10.1371/journal.pone.0191649 (PMC5796695; doi:10.1371/journal.pone.0191649)
Supplement: S1 Appendix — (PDF) [file pone.0191649.s001.pdf]

Supporting Information 1. Simulation single-rule case

We start by defining some terminology (Laver and Sergenti 2012). A *model run* is a simulation with distinct parameter input (e.g., a three-party system or a four-party system). *Repetitions* are simulations that have the same input on all core variables (i.e., same model run) but use different seeds for the quasi-random number generator. Finally, an *iteration* is a single step in a repetition in which each party applies its decision rule once. Usually, a repetition is composed of several iterations.

Simulation strategy and mean estimation

We now turn to describing our simulation strategies. For the following technicalities, our major interest lies in the state variable party system eccentricity. Mathematically, the party systems that we simulate can be represented as Markov chains and, hence, the varying nature of policy eccentricity is represented by the state space. As usual for Markov chains, we have to determine the number of iterations it takes the chain to enter a steady stage distribution. Since Stickers do not move once they are initiated, they are burnt-in immediately. For all other decision rules, we ran model runs with parameters for which we expect the burn-in phase to take the longest.[1] To rule out that the burn-in time we determine is biased by accidently drawing an unusual repetition, we run three repetitions per model run. As soon as a repetition's party system eccentricity varies in a regular form or ceases to vary at all, it is burnt in. Even though burn in length varies by decision rule, we opt for consistency and run 75 burn iterations for all rules. Only for Hunters, burn-in is 100 iterations.

Table A: Mean Estimation Strategies

Rule	Number In Code	Burn-In Iterations	Saved Iterations	Number of Repetitions	Average Type
Sticker	4	75	1	500	Ensemble
Governator	3	75	1	500	Ensemble
Hunting Gov	8	75	1	500	Ensemble
Hunter	7	100	500	1	Time
Aggregator	2	75	1	500	Ensemble

Once a repetition reached a steady state, we could theoretically map out the entire state space of party system eccentricity, however, this would be computationally too demanding. Instead we rely on the mean party system eccentricity in steady state (Laver and Sergenti 2012, 66-68). We use two different estimation strategies to obtain reliable estimates of this quantity: Except for the Hunter, we compute an ensemble average for all decision rules. In particular, we

[1] We are not sure which parameters produce the longest burn-in period. Therefore, we test 16 parameter combinations (i.e., 16 model runs) for each decision rule. These combinations are composed of all possible combinations for: $\alpha \in \{0,1\}, discount \in \{0,1\}, and\ Ideal\ Point\ Dispersion\ Factor \in \{0, 2\}$.

run 500 repetitions of each model run until they are burnt-in.2 After having finished the burn-in iterations, we run each repetition for one more iteration and save the party system's eccentricity. Our estimate of a model run's party system eccentricity (and the other variables of interest) is the average of all of its repetitions' party system eccentricities at the first iteration after burn-in. For Hunters, we compute a time average (Laver and Sergenti 2012, 88-90) of 500 post-burn-in iterations of a single repetition per model run.[3] Table A summarizes these values.

Assessing parameter impact

We analyze how party system eccentricity, system misery, and government misery vary with input parameters. Even though we simulated only certain values of the parameters, we can use regression techniques to make predictions about all scenarios. Since we know which parameters vary among model runs, we are mainly interested in getting the functional form and interactions of parameters right.

Because we have almost no theoretical expectations how a certain parameter shapes the quantities of interest,[4] we rely on statistical tools designed to uncover the "correct" functional form of covariates in a regression framework. In particular, we draw upon Kenkel and Signorino's implementation of the adaptive LASSO method: polywog (Kenkel and Signorino 2011, 2013). Simply put, this approach tests various interactions and polynomials of pre-specified independent variables and selects the model with the best fit given certain model complexity constraints. We model each decision rule's variables of interest as a function of the model parameters, leaving the exact functional form open for assessment by the polywog procedure.

We run separate regressions for each rule and each model output average (i.e., eccentricity, party system misery, and government misery). We let polywog estimate a functional form,[5] compute corresponding standard errors on coefficients using the non-parametric bootstrap procedure 400 times, and remove all terms from the equation that are not statistically differentiable from having no effect at the 95% confidence-level. The remaining terms are modeled again in an OLS regression. Overall, our models provide an outstanding model fit and manage to capture the data generating process well.

---

[2] We find that using the means of 500 repetitions ensures that virtually all mean party system eccentricities we compute are within the 95% confidence interval of a mean party system eccentricity computed from 2000 repetitions. This is a conservative, yet, computationally not too demanding solution.

[3] We find that already after about 200 iterations, the time average moves within a standard deviation of the estimated mean system eccentricity of 2000 iterations. To make results comparable to other rules, we estimate an average of 500 iteration though.

[4] In some case, though, we have theoretical expectations. Stickers, for instance, should not respond to any incentives with respect to eccentricity. Also, Aggregators will converge to an optimal representation of the space (Lloyd 1982).

[5] The polywog procedure requires that the researcher specifies a maximal degree of complexity for the regression equations. In particular, one needs to decide how many factors may be interacted, including identical factors to obtain powers. Using ten-fold cross validation, we estimate the cross-validation error of each degree of complexity (maximally five-fold interaction) and choose the model that minimizes cross-validation error.
