# Supplementary material for: Governator vs. Hunter and Aggregator: A simulation of party competition with vote-seeking and office-seeking rules
Source: PLoS One. 2018 Feb 2;13(2):e0191649. doi: 10.1371/journal.pone.0191649 (PMC5796695; doi:10.1371/journal.pone.0191649)
Supplement: S2 Appendix — (PDF) [file pone.0191649.s002.pdf]

Supporting Information 2. Effect of Discounting the Caretaker Government

We discuss how discounting caretaker governments matters (see Figure A). Discounting does not matter too much if parties value policy (right panel). If they don't, however, increasing discounting of caretaker governments improves government representation substantially. The exceptions from the rule are Governators. They worsen the situation as discounting increases. This is because all parties converge to one position, the government position.

Figure A. Effect of Discount Factor for Caretaker Governments

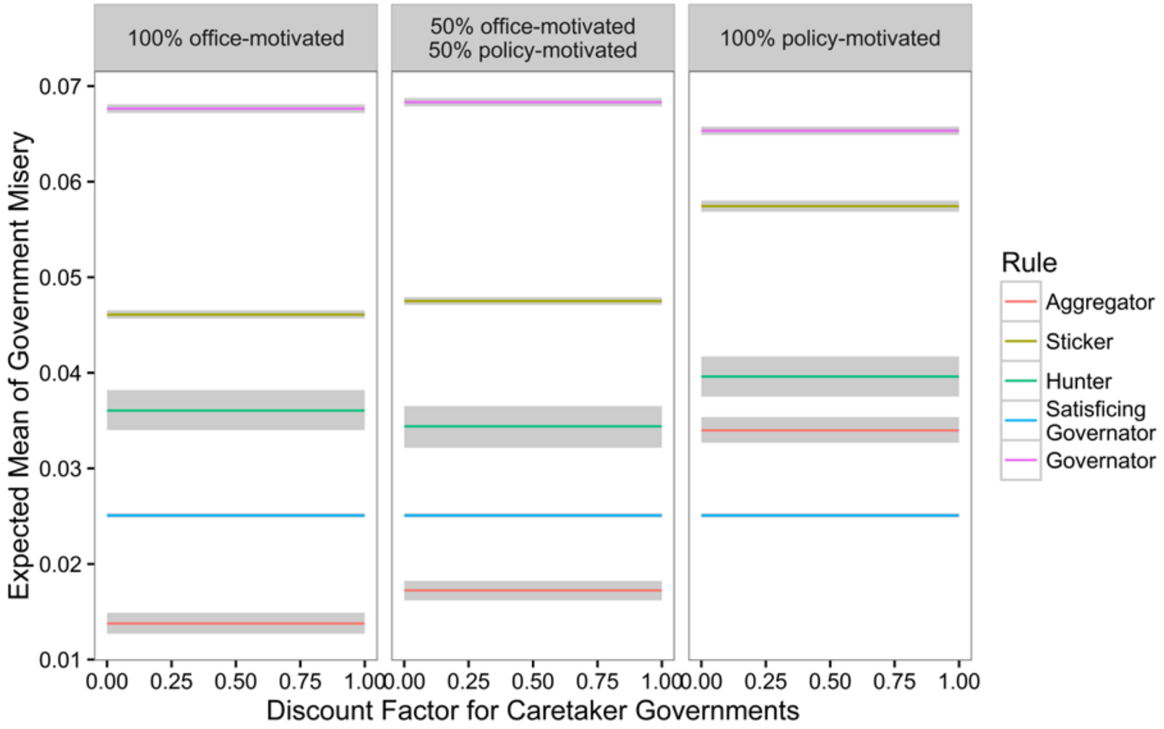

Note: Based on corresponding data mined OLS regressions with 5 parties, and ideal point variance factor at 1. Grey shaded areas are 95% confidence intervals. Columns represent different levels of policy-motivation.
