# Supplementary material for: Governator vs. Hunter and Aggregator: A simulation of party competition with vote-seeking and office-seeking rules
Source: PLoS One. 2018 Feb 2;13(2):e0191649. doi: 10.1371/journal.pone.0191649 (PMC5796695; doi:10.1371/journal.pone.0191649)
Supplement: S3 Appendix — (PDF) [file pone.0191649.s003.pdf]

Supporting Information 3. Government characteristics in non-evolutionary model

The model also provides information on governments that are formed and how these change as different decision rules are used or model parameters are varied. We focus our attention on the following aspects:

- the number of parties that form a government,
- whether a majority of MPs belong to the government parties,
- the share of seats government parties control in parliament,
- the ideological cohesion of government parties,
- whether a government member party can leave the government without the government losing its majority in parliament (oversized government).

The most striking observation for government formation is that vote-oriented rules form different governments than office-oriented rules. Recall that the actual procedure that parties follow to form governments does *not* vary across decision rules – only their decision when or how to shift policy positions varies. In fact, it is the party-system the decision rules gives rise to which determines how governments are formed.

Since office-oriented rules make parties' converge to similar, centrist positions, the inclusion of additional parties to the government does neither change policy much nor the distribution of government seats. Hence, they form large majorities, many times even oversized, governments that nevertheless are more cohesive than vote-seeking rules' governments. In fact, Governators make all parties converge to a single policy-position which makes government cohesion perfect, yet, at positions that on average hurt voter representation by the government. The top panels of Figure A clarifies this point.

Another interesting finding is that extreme party systems, i.e., party systems without discounting of caretaker governments, or with solely policy-motivated or solely office-motivated parties stand out. Figure A shows this effect for government cohesion (top row), the share of majority governments (center row), and the effective number of parties in government (bottom row) as a varying number of parties compete in an election (x-axis) for different levels of policy-motivation (columns). While all rules see a similar increase in ideological differences as the number of competing parties increases and parties are fully office-motivated, Satisficing Governators manage to form more ideologically cohesive governments than other rules when policy matters.[1] This pattern is rooted in the fact that more parties are linked to more eccentricity for votes-oriented rules and less eccentricity for office-oriented rules. Hence, additional government members that are needed to form a viable government, will shift the government – on average – in the corresponding direction.

[1] Again, Governators make all parties converge to a single position.

Figure A. Government formation in simulations with different rules

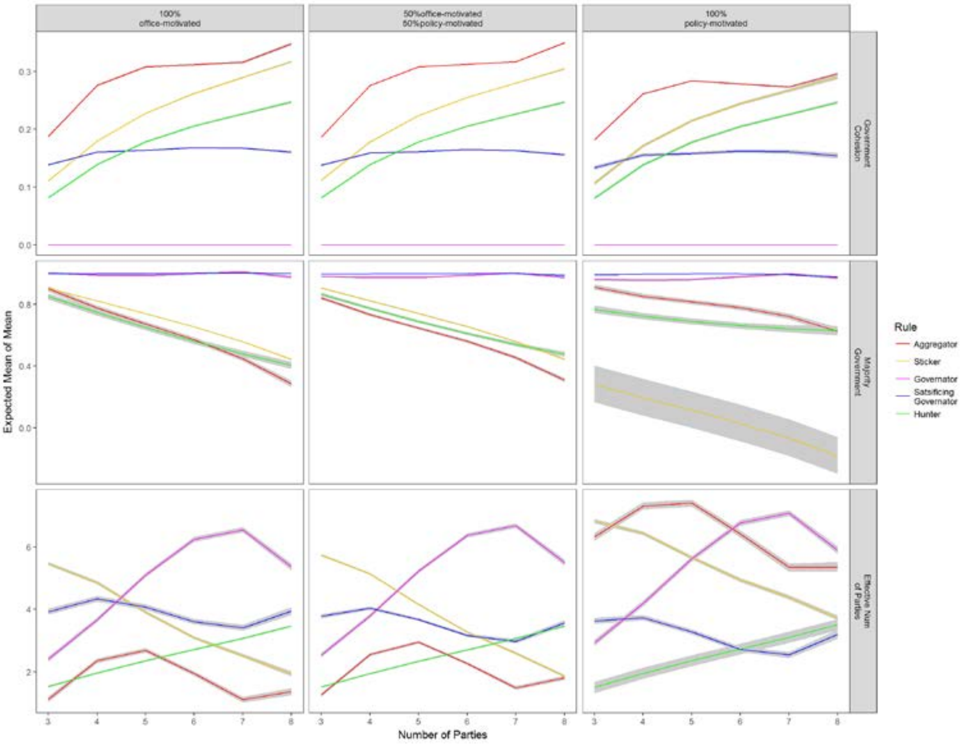

Note: Based on corresponding data mined OLS regressions with standard ideal point variance, and discount factor = .5. Grey shaded areas are 95% confidence intervals. Columns represent different levels of policy-motivation.

A similar pattern is shown in the center row of Figure A. It displays the share of majority governments that are formed. As long as policy does not matter at all (left column), all rules tend to form majority governments only. As the bottom panel reveals, this fact comes with the expected increase in the effective number of government parties. The center columns displays the significant changes of mixed party motivations: Vote-oriented rules tend to form fewer majority governments as the number of parties increase (center panel), yet, the number of effective government parties increases (center bottom panel). Office-motivated parties, by contrast, stick to the high level of majority governments. Minority governments formed by government-oriented rules occur frequently only when parties are purely policy-seeking.

These contradictory patterns in all three, policy position taking, government formation, and patterns of representation raise the question how mixtures of these rules perform when competing with one another.
