# Supplementary material for: Governator vs. Hunter and Aggregator: A simulation of party competition with vote-seeking and office-seeking rules
Source: PLoS One. 2018 Feb 2;13(2):e0191649. doi: 10.1371/journal.pone.0191649 (PMC5796695; doi:10.1371/journal.pone.0191649)
Supplement: S4 Appendix — (PDF) [file pone.0191649.s004.pdf]

Supporting Information 4. Burn-in length for evolutionary simulation.

We need to determine three burn-in lengths:

1) Time it takes the model to burn in before the evolution part starts. This takes 75 iterations.

2) Time to burn in once the evolution has started. This takes 1000 iterations.

3) How many repetitions are needed to get a representative ensemble average of the steady state. This takes 500 iterations.

Stage 1:
We ran 32 model runs with parameters that we suspected are linked to long burn-in. These are the same parameters as in the single-rule simulation (the minimum and maximum values of memory, discount and ideal point dispersion). We ran 500 repetitions of each model runs. Each model run runs for 5000 iterations. Using graphs and the Effective Number of Rules (ENR) in the system (Laver and Sergenti 2012), we find that after 75 iterations the model run is burnt in.

Stage 2:
We now reduce the input grid to all minimum and maximum values of discount, memory, aspiration, alpha, and ideal point dispersion. There are 10 repetitions of each model run. A repetition starts with 75 non-evolution iterations, then 2000 evolution iterations follow. Again, using the same graphical tools and ENR as state variable, we decide that the repetitions are burnt in after 1000 iterations.

Stage 3:
We use the same model runs as before. Both burn-ins are applied as just described. Each model runs has 2000 repetitions of which we keep the 1076st iteration. We are now interested in getting eccentricity, representativeness, and government representation right. Assuming that the ensemble mean of 2000 of these is the "true" mean, we compute the ensemble mean of 100, 200, 300 repetitions and so on. We find that the mean of 500 repetitions is a good but not perfect proxy. It is usually, but not always, within the 95% confidence intervals of these means.
