# Supplementary material for: Governator vs. Hunter and Aggregator: A simulation of party competition with vote-seeking and office-seeking rules
Source: PLoS One. 2018 Feb 2;13(2):e0191649. doi: 10.1371/journal.pone.0191649 (PMC5796695; doi:10.1371/journal.pone.0191649)
Supplement: S5 Appendix — (PDF) [file pone.0191649.s005.pdf]

Supporting Information 5. Correlation between rule occurence in party systems

Table A. Correlations of Ensemble Averages of Rule Shares across all Model Runs.

	Aggregator	Governator	Sticker	Hunter
Aggregator				
Governator	-.83			
Sticker	-.03	-.10		
Hunter	.76	-.86	-.05	
Satisficing Governator	-.84	.66	-.20	-.82

All correlations are significant with $p<.05$.
