# Supplementary material for: Governator vs. Hunter and Aggregator: A simulation of party competition with vote-seeking and office-seeking rules
Source: PLoS One. 2018 Feb 2;13(2):e0191649. doi: 10.1371/journal.pone.0191649 (PMC5796695; doi:10.1371/journal.pone.0191649)
Supplement: S6 Appendix — (PDF) [file pone.0191649.s006.pdf]

Supporting Information 6. Analysis of rule change

What factors make parties change their rules more often? And which factors drive the number of rules a party uses? Given that parties choose a new rule randomly, the answers to these questions are strongly correlated. In fact, plots of these figures look virtually identical (i.e., whenever the rate of rule change is high, parties use, on average, many rules), and we hence focus on the rate of rule changes here. To detect rule changes, we extend the period we trace parties' choices after burn-in from one iteration to 100 iterations.

By design, the memory parameter controls how often parties can change rules within the observed period. For example, if parties can change rules every other iteration ($m=2$), the average share of rule changes per round has a theoretical maximum at .5. Put differently, if every party changes rules every m rounds, we would expect a share of $1/m$ parties to change rules each round. In below figures, we depict this theoretical maximum as red lines. Also, we do not plot results of the data mining procedures used before, yet black lines are loess estimates.

The left panel of Figure 1 depicts the expected mean share of rule changes per iteration in a four–party system with fully office-motivated parties and the aspiration level set at .5. Put differently, unless a party wins a majority of government seats, it cannot meet the aspiration level and will change rules whenever possible. The way the expected rate of rule changes traces the expected maximum rate of rule changes, indicates that this actually happens.

Figure 1's center panel shows that when parties are equally policy- and office-motivated, the share of rule changes drops significantly, yet, its shape still resembles the expected maximum of rule changes. This happens because opposition parties change rules frequently, yet government parties don't. Finally, if parties do not care about office spoils at all, they hardly change rules in steady state because governments form that are centrist enough to satisfy all parties.

Figure 1. Expected Mean Share of Rule Changes with varying memory and policy-motivation

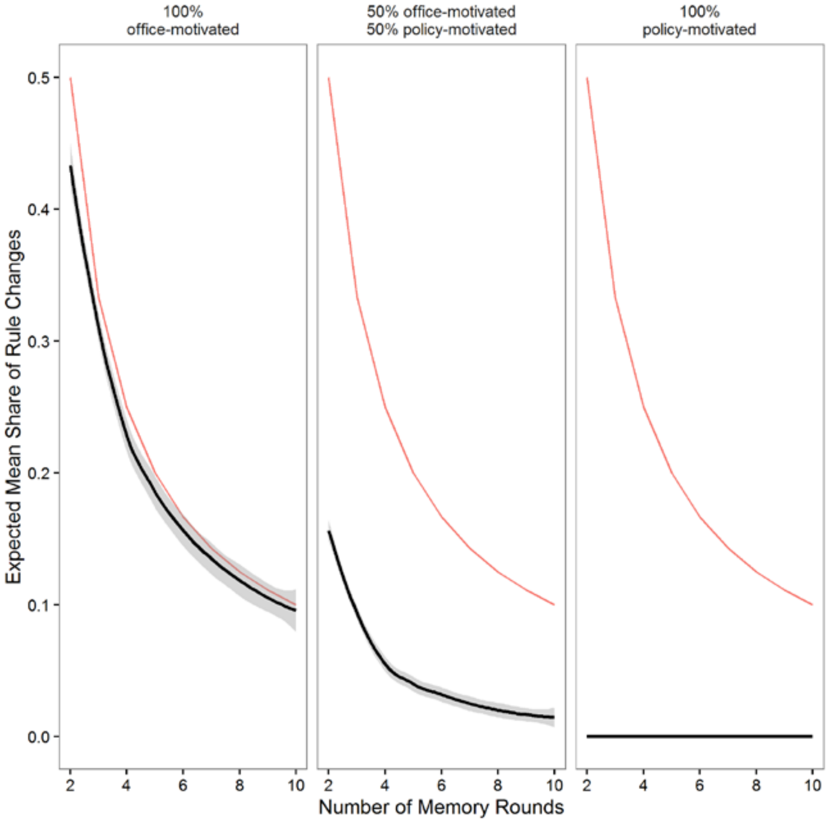

Note: Loess predictions with standard ideal point variance, four parties, discount factor at .5, and aspiration at .5. Grey shaded areas are 95% confidence intervals. Columns represent different levels of policy-motivation. Red lines indicate maximal possible rule change.

Figure 2 depicts the substantial significance of the level of policy-motivation that exhibits the same pattern as described above.

Figure 2. Expected Mean Share of Rule Changes as Policy-Motivation Varies

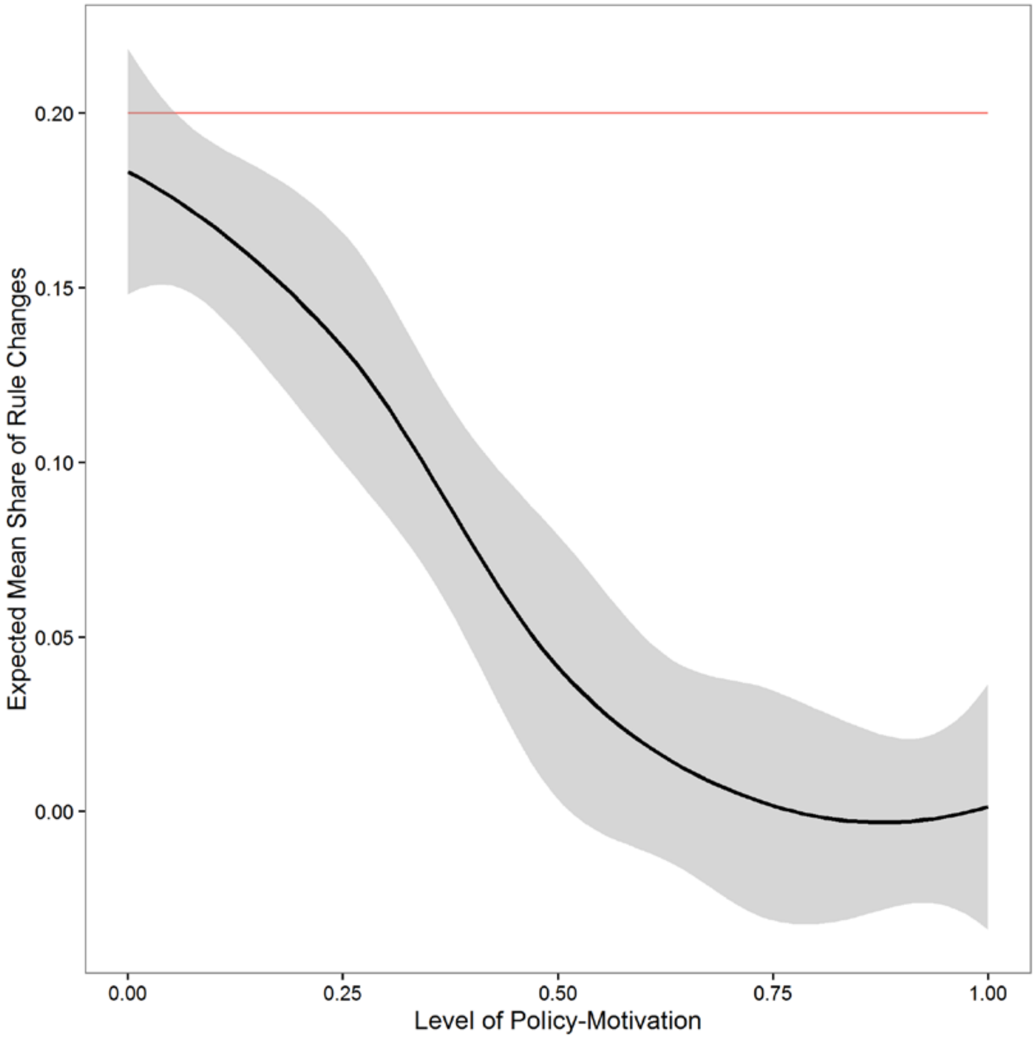

Note: Loess predictions with standard ideal point variance, four parties, discount factor at .5, five iterations of memory, and aspiration at .5. Grey shaded areas are 95% confidence intervals. The red line indicates expected theoretical maximal rule change.

The level of policy-motivation does not only condition the effect of the length of party memory, yet, is also mitigates the effect of the number of parties on expected mean rule changes as Figure 3 shows.

Figure 3. Expected Mean Share of Rule Changes as Number of Parties and Policy-Motivation Vary

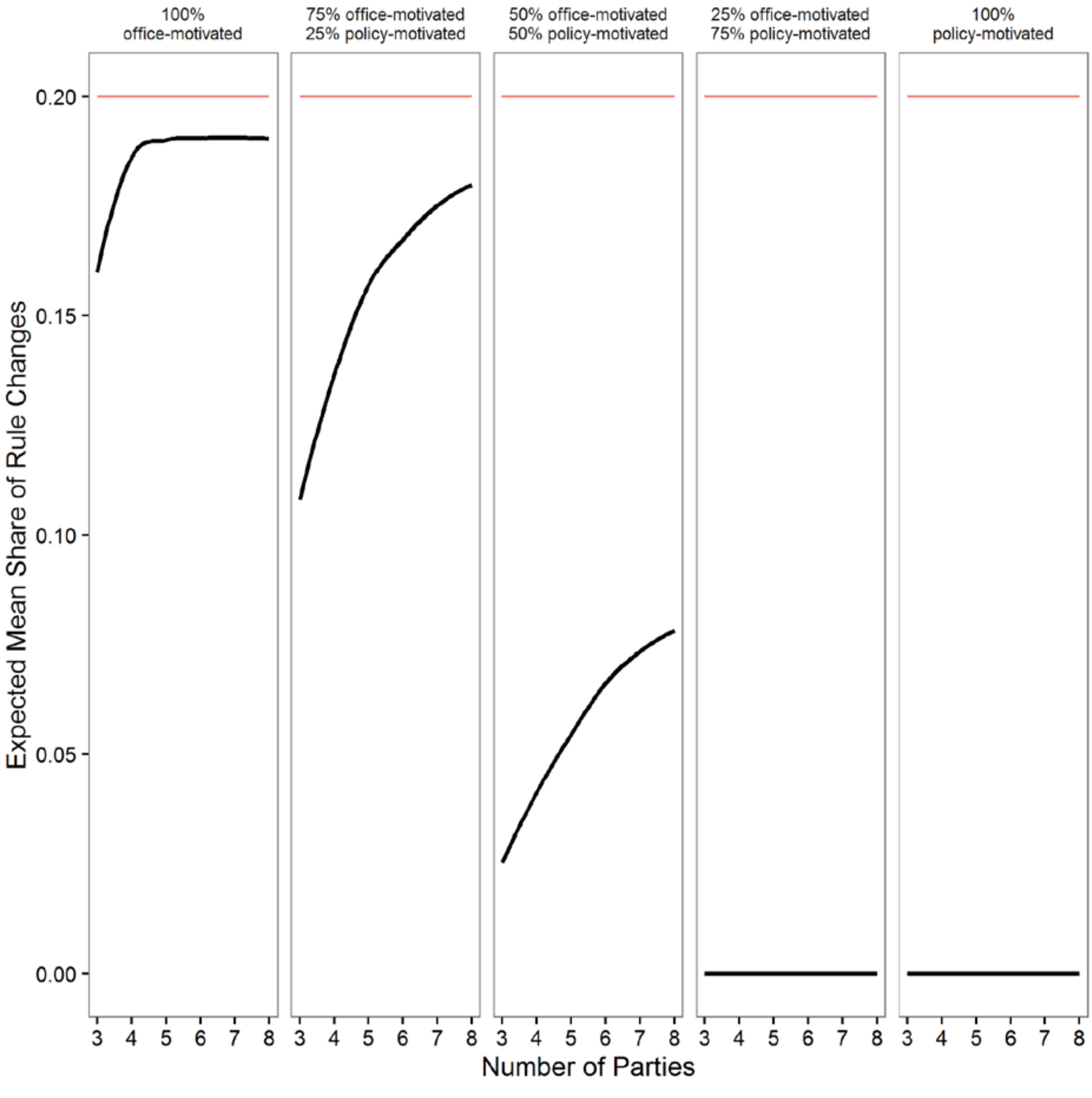

Note: Loess predictions with standard ideal point variance, four parties, discount factor at .5, five iterations of memory, and aspiration at .5. Grey shaded areas are 95% confidence intervals. Columns represent different levels of policy-motivation. Red lines indicate maximal possible rule change.

When parties are mostly or solely office-seeking, virtually all parties change rules whenever possible, and the share increases in parties since it becomes harder to former single-party governments. The overall rate of rule changes decreases as policy becomes more valuable to parties. Eventually, when parties' policy-motivation dominates they no longer change rules at all.

Figure 4. Expected Mean Share of Rule Changes as Level of Aspiration and Policy-Motivation Vary

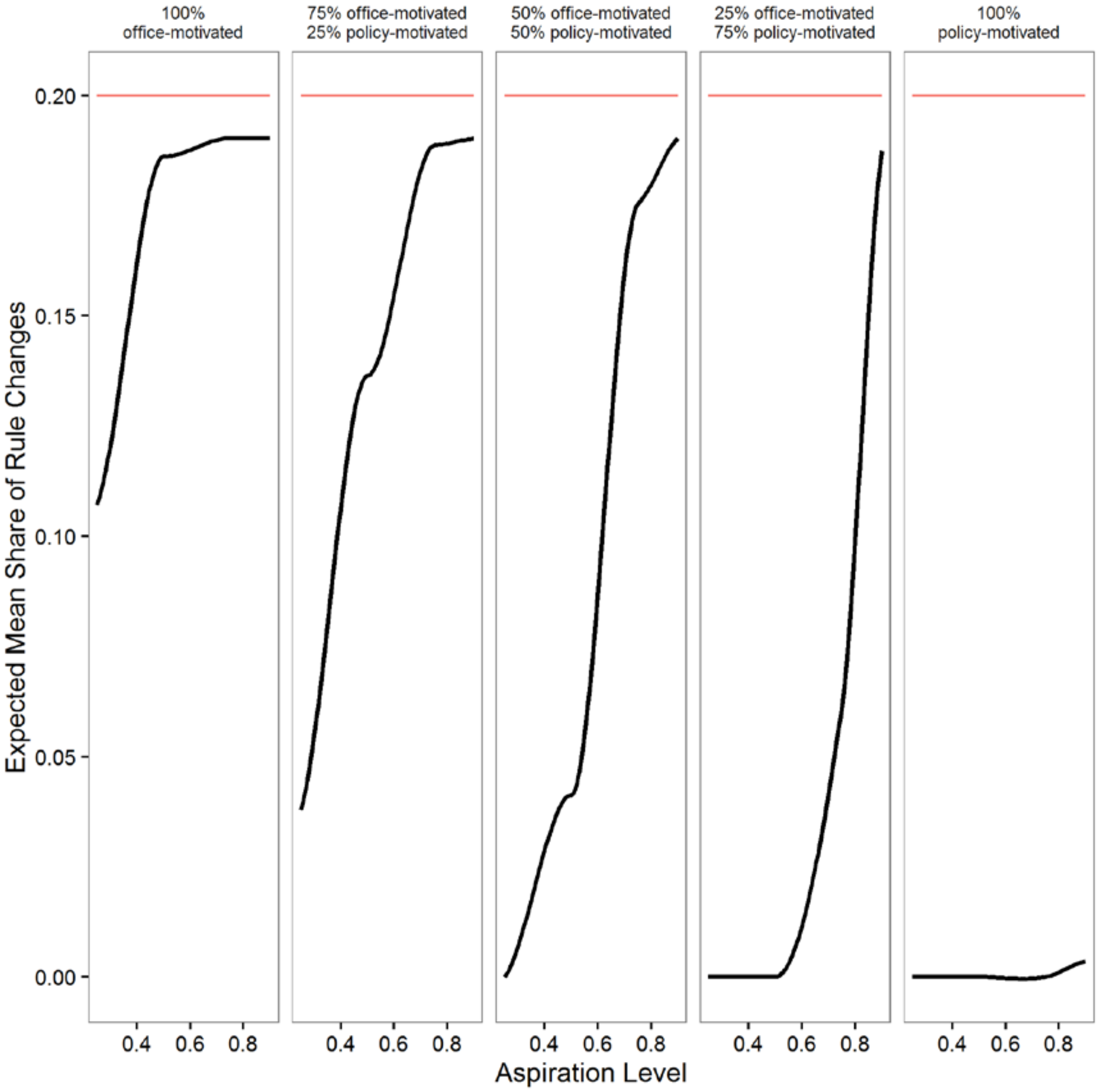

Note: Loess predictions with standard ideal point variance, four parties, discount factor at .5, and five iterations of memory. Grey shaded areas are 95% confidence intervals. Columns represent different levels of policy-motivation. Red lines indicate maximal possible rule change.

Aspiration, again, matters at certain values of policy-motivation. As before, when only office yields utility, rates of rule change are high. Of course, they further increase in the level of aspiration. Once policy contributes a significant share of utility, rates of rule change drop significantly for low levels of aspiration. Yet, for very high levels of aspirations, the rate of rule changes is always high. When office does not matter for utility, aspiration does not matter much for the share of rule changes unless aspiration is very high. This is rooted in the fact that centrist governments allow virtually all parties to be happy if only policy matters.

The discount factor as well as the dispersion of parties' ideal positions does not substantively matter for the rate of rule changes.
