# Supplementary material for: Governator vs. Hunter and Aggregator: A simulation of party competition with vote-seeking and office-seeking rules
Source: PLoS One. 2018 Feb 2;13(2):e0191649. doi: 10.1371/journal.pone.0191649 (PMC5796695; doi:10.1371/journal.pone.0191649)
Supplement: S7 Appendix — (PDF) [file pone.0191649.s007.pdf]

Supporting Information 7. ABM-ODD protocol

Purpose
The purpose of the model is to understand what decision rules give rise to party system misery and government misery.

Entities, state variables, and scales
The model's basic entities are political parties. Each party has state variables ideal policy position, stated policy position, government membership, the decision rule it uses to adjust its stated policy position, its level of policy-seekingness, and the discount factor for utility provided by caretaker governments. Parties adjust their positions given its decision rule and form governments given the state variables and election results. In the evolutionary version of the model, parties also have state variables memory (i.e., the number of rounds a party can recall) and the aspiration level which needs to be met for a party to not change its decision rule.

The policy space is comprised of two policy dimensions (Laver 2005), each of length $\sqrt{2}/2$ in order to simplify computation and limit maximal policy loss at 1 (see section on parties' utility function). The decision rules are clarified below. The level of policy-seekingness as well as the discount factor are bounded between 0 and 1 as these are the logical limits to both variables. Memory is any integer between 2 and 10 rounds. Aspiration can take values .25, .5, .75, or .9 because these balance the need for a small sample space and theoretically interesting values.

Scheduling
Each iteration begins with parties updating their stated policy position using their decision rule. Subsequently, they learn their electoral support (voters vote for the party closest to them, ties are broken randomly) and form a government. In the evolutionary model, parties may change their decision rule.

Design concepts
The model relies on the spatial modeling literature in political science (Downs 1957), however contends that actors act in an environment of uncertainty that impedes their strategic behavior significantly (Budge 1994; Laver 2005). To cope, parties use heuristics to make decisions. At the system-level parties' choices, thus, give rise to different position compositions which affect how well the party system represents voters' interests. Moreover, parties form governments that may represent voters' interests more or less well.

We collect data on party system misery and government misery.

Input data
Several model variables function as preset model input even though they are randomly generated. This is necessary to compute valid ensemble averages (see Supporting Information A). In a nutshell, random input parameters are created and simulated with different seeds for the random number generating procedure. These preset variables are the number of parties (any integer in [3, 8]), the dispersion of their ideal policy positions (any number in [0, 2]), the discount factor for caretaker governments (any number in [0, 1]), their level of policy-seekingness (any number in [0, 1]). In the evolutionary version of the model, this also includes parties' aspiration level (.25, .5, .75, or .9) and the length of their memory (any integer in [2, 10]).

Initiation

To initialize, parties' positions in both dimensions are randomly drawn from a normal distribution truncated at 0 and $\sqrt{2}/2$. The distribution has its mean at $\sqrt{2}/4$, its standard deviation and the number of parties drawn are given by model input. The positions that are drawn are considered to be parties' ideal positions. A random sample of these parties are made government parties. Also, 1000 voters' ideal positions in both dimensions are randomly drawn from the same truncated normal distribution as above, yet, the standard deviation is set to .12. All other variables are given by model input.

Sub models

Government policy position:

The government policy position is the weighted mean of government parties' stated policy positions on the corresponding policy dimensions with government parties' seat shares of the sum of all government parties' seats as weights.

Party utility:

$$U_t^k = \alpha \times p_t^k + (1 - \alpha) \times s_t^k$$

Where *k* is the superscript for a particular party, *t* denotes the time period, α is the level of policy-seekingness the party has, *p* is 1 minus the squared Euclidean distance between the ideal party policy position and the government position, and *s* is the share of seats the party contributes to the sum of all government party seats.

Decision rules:

Aggregator (Laver and Sergenti 2012, 139): Identify the mean coordinate on each dimension of the ideal points of your current party supporters; move in this direction unless this causes you to overshoot, in which case move to the mean of supporter ideal point.[1]

Hunter (Laver and Sergenti 2012, 45): If previous move led to fewer votes, reverse direction and move on a heading randomly selected within the half-space now being faced. Otherwise, move in the same directions as last round.

Sticker (Laver and Sergenti 2012, 43): Stay put.

Governator: Move toward the government position unless this causes you to overshoot, in which case move to the government position.

Satisficing Governator: If member of the current government, stay put. Otherwise, move toward the government position unless this causes you to overshoot, in which case move to the government position.

Government formation procedure:

1. Parties compute the utility they would obtain from all potential governments – including governments they do not participate in, oversized and minority governments.[2]
2. Parties rank all of these governments according to their utility. They report their ranking to a non-partisan head of state who will suggest a government to the parties in step four.
3. The head of state weighs the preferences revealed to her by parties' parliamentary seat shares. This means that large parties' preferences will be more influential.
4. The head of state suggests the highest ranked government to parties.[3]
5. Each party compares the utility it receives from the suggested government to the scenario in which the current government remains in office as a caretaker government.

[1] If a party is allowed to move maximally .12 units but its target position is only .1 units away, then it would overshoot if it moved by .12 units.

[2] We assume that parties without parliamentary representation cannot participate in governments.

[3] Ties between governments are broken at random.

Since caretaker governments cannot pass legislation as easily as ordinary governments, the utility parties receive from caretaker governments are discounted by a factor which is a model parameter we vary. If the suggested government yields at least as much utility as the lurking caretaker government, a party signals its support for the candidate government to the head of state.

6. Having learned parties' support for the candidate government, the head of state evaluates whether the candidate government is supported by (1) all members of the candidate government and (2) a majority of the parliament. If either of these conditions is not met, the head of state repeats step 4 (i.e., suggesting the highest ranked government to parties), yet, she erases the just suggested candidate government from her list. This procedure continues until either a candidate government is supported by all of its members and a majority in parliament, or all candidate governments are rejected and the caretaker government is installed.

Rule evolution:

Let *k* be a party with memory *m* and aspiration level *a*. The evolution rule is:

1. If party *k* has used the same rule in the last *m* rounds, compute *k*'s mean utility of the last *m* rounds, U_m^k.

2. If $U_m^k < a$, choose a new decision rule randomly.
