# Supplementary material for: Governator vs. Hunter and Aggregator: A simulation of party competition with vote-seeking and office-seeking rules
Source: PLoS One. 2018 Feb 2;13(2):e0191649. doi: 10.1371/journal.pone.0191649 (PMC5796695; doi:10.1371/journal.pone.0191649)
Supplement: S8 Appendix — (PDF) [file pone.0191649.s008.pdf]

Supporting Information 8. Variability analysis

The analyses in the main text are centered on mean analyses, i.e., on average what consequences do certain combinations of model parameters and decision rules have on party system misery or government misery? In this section, we investigate how these affect the variability of results. To do so, we apply our data mining procedure (see Supporting Information 1), yet, we do not evaluate the mean of a variable of interests over models runs (or within a model run for Hunter-only models). Instead, we analyze their standard deviations.

Single-rule simulations

As Figure A indicates, decision rules have differing levels of variability in their performances. Overall, the expected variability (i.e., standard deviation) in simulation results is moderate and in most cases less than half the size of the expected means. Variability in government misery is somewhat higher, yet, even in extreme circumstances substantively smaller than the expected mean.

Figure A. Expected Variability and Party System Size

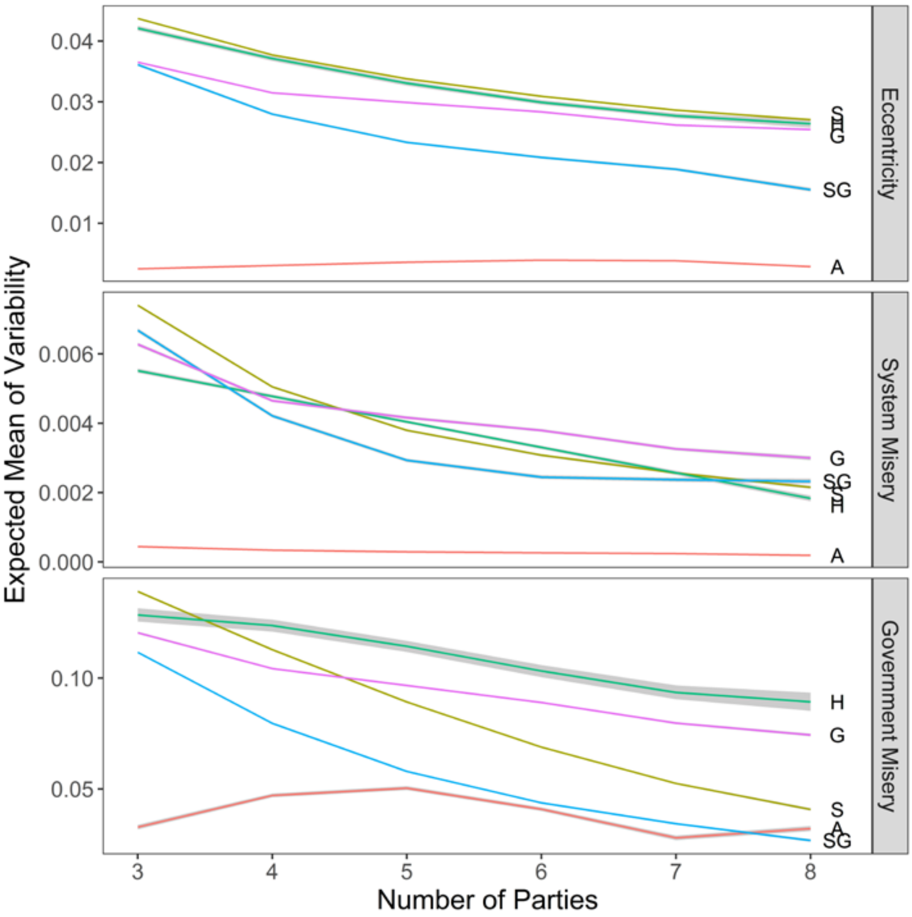

Note: Based on corresponding data mined OLS regressions with ideal point variance factor at 1, discount factor at .5 and policy motivation at .5. Grey shaded areas are 95% confidence intervals. A = Aggregator, S = Sticker, H = Hunter, G = Governator, SG = Satisficing Governator.

In terms of individual decision rules, Aggregators variability is very low which is because they spread out in a very similar way irrespective of model parameters. Consequently, their variability in terms of party system misery and government misery is always fairly low. The other decision rules tend to have similar variability that is clearly higher than Aggregators' variability. Among these rules, Satisficing Governators seem to be the lowest variability rule. Figure A further shows that as the number of parties in a party system increases, variability in performance decreases for all decision rules. This is because more parties are less likely to be initiated in a certain region of the policy space only, leading to more stable results over time.

Figure B. Expected Variability and Policy-Seekingness

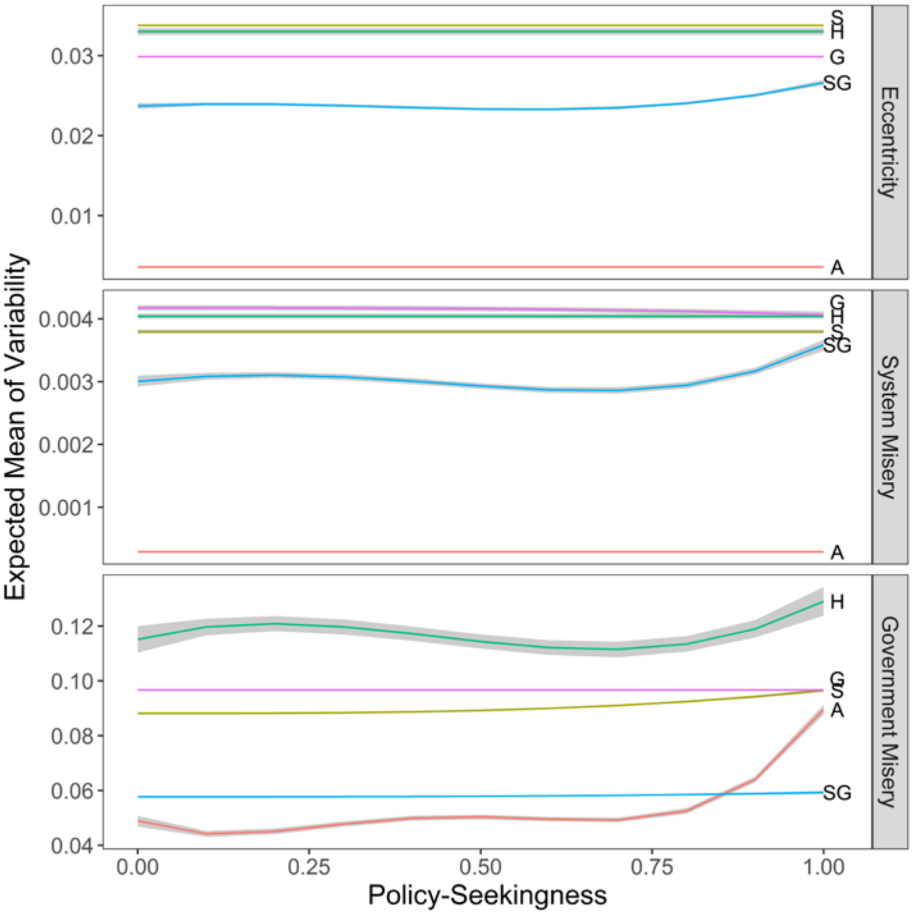

Note: Based on corresponding data mined OLS regressions with ideal point variance factor = 1, five parties and discount factor at .5. Grey shaded areas are 95% confidence intervals. A = Aggregator, S = Sticker, H = Hunter, G = Governator, SG = Satisficing Governator.

Figure B reveals that policy-seekingness affects variability at high levels only. That is, as long as parties' utility is composed of at least 25% of office payoffs, variability is not influenced. As parties become more policy-motivated, however, variability increases substantially because government formation (and thereby for Governators their point of orientation for policy shifts) is more likely to be affected by the individual set of party positions in a given simulation. The reason why sufficiently high office payoffs impede this effect is that they enable governments be to more ideologically divers, leading to more centrist and less variable outcomes.

Figure C. Expected Variability and Ideal Point Dispersion

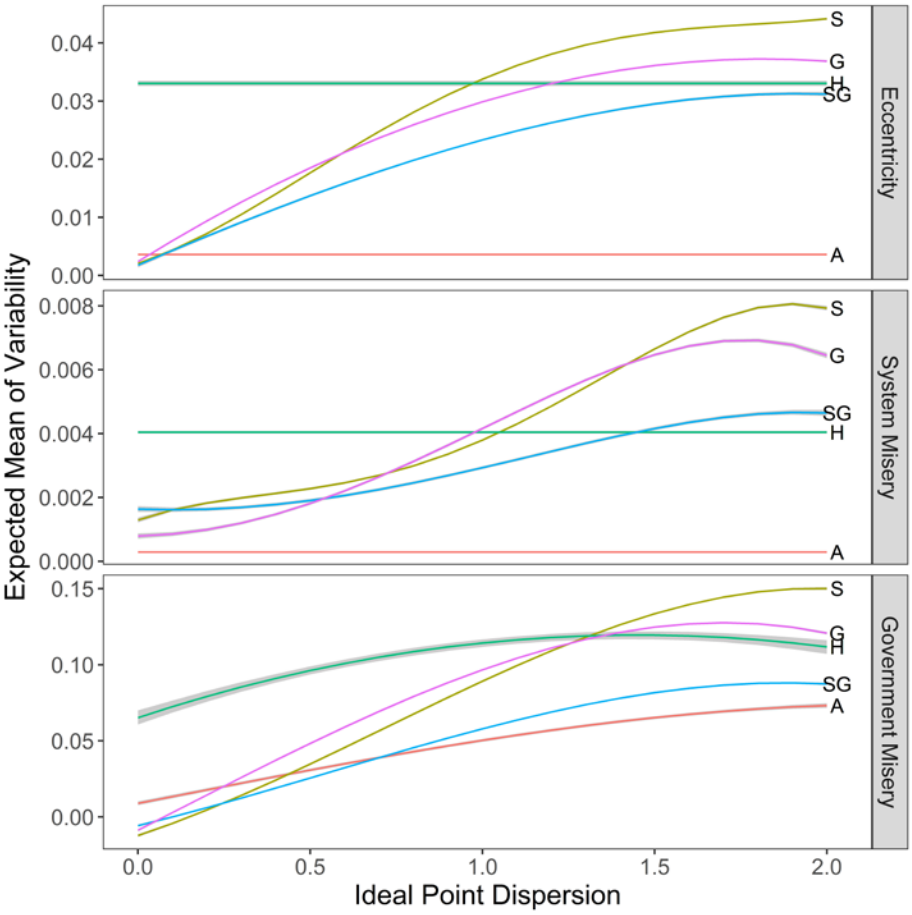

Note: Based on corresponding data mined OLS regressions with policy-seekingness at .5, five parties and discount factor at .5. Grey shaded areas are 95% confidence intervals. A = Aggregator, S = Sticker, H = Hunter, G = Governator, SG = Satisficing Governator.

Figure C depicts the relationship between variability and the dispersion of parties' ideal points. As Aggregators and Hunter decide on their policy positions without any influence of government formation and hence ideal points, their variability for eccentricity and system misery is not affected by ideal point dispersion. All other rules, for which ideal point dispersion is the only determinant of eccentricity (Sticker) or a major factor due to government formation (both types of Goverators), increase variability in eccentricity and therefore variability in system misery as ideal point dispersion increases. For all rules, variability in government misery increases with ideal point dispersion because parties take more extreme policy ideal points, and again more variable government outcomes are likely.

The discount factor of caretaker government does not have an effect on variability of the quantities of interest.

Evolutionary simulations

In general, the evolutionary variability is most determined by the rules competing. We, therefore, focus our attention to rule shares.

Figure D. Expected Variability in Evolutionary Simulations

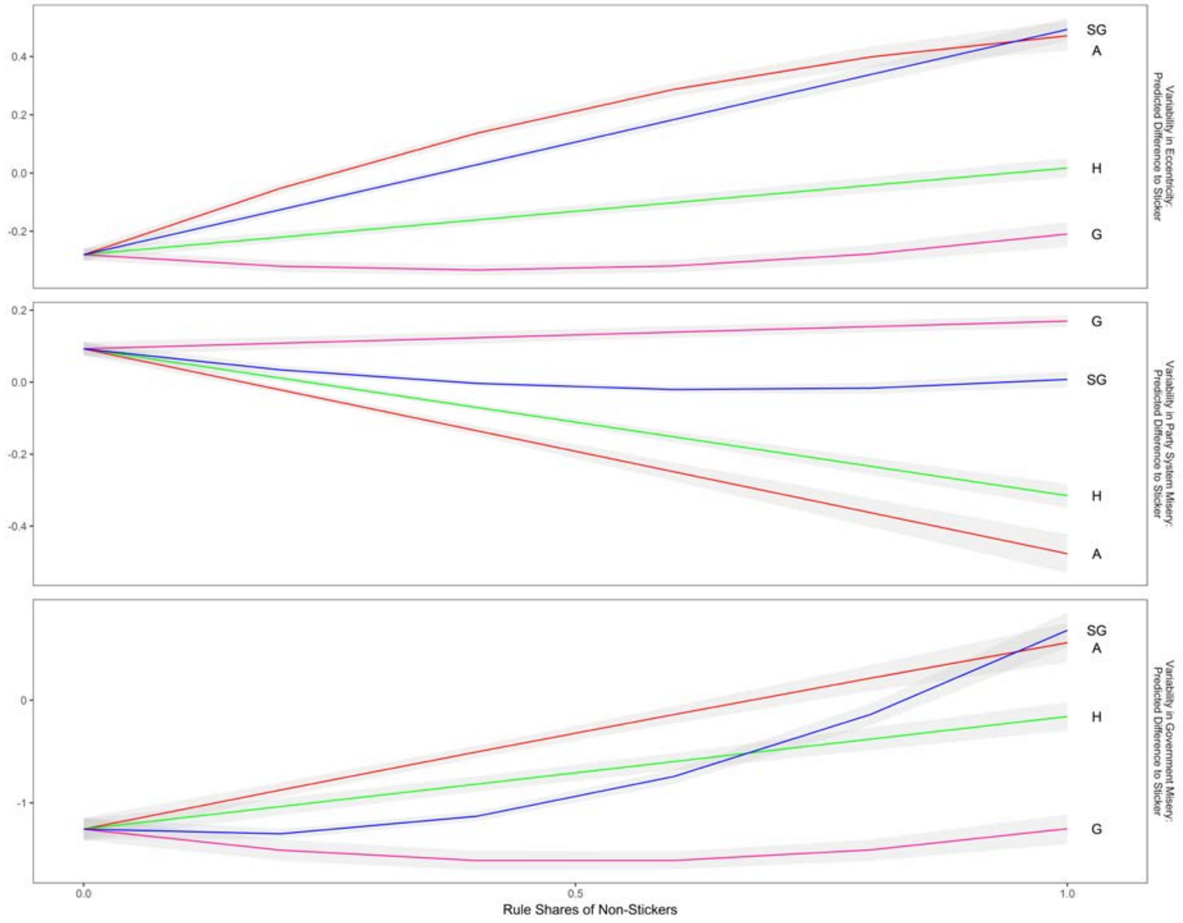

Note: Based on corresponding data mined OLS regressions with policy-seekingness at .5, five parties, ideal point dispersion at 1 and discount factor at .5. Grey shaded areas are 95% confidence intervals. Stickers are the reference category. A = Aggregator, S = Sticker, H = Hunter, G = Governator, SG = Satisficing Governator.

The results for variability in eccentricity (top panel of Figure D), again, indicate that variability is not too high relative to eccentricity. Interestingly, variability in eccentricity becomes greater as many Aggregators or Satisficing Governators compete in a party system. Hunters and Governators lead to less variability than Stickers (reference category).

At the same time, the variability in party system misery (center panel) decreases as many Aggregators compete. This is due to their specific property to spread out equally across the policy space. While it is not a safe bet to predict where Aggregators locate, it is a safe bet to predict that they will decrease party system misery (see also main text). Hunters have a similar property, the safe bet here is for bad representation and high government misery though. The high variability of Governators is translated into high variability of party system misery because their point of orientation, i.e., the government position, is highly variable.

In terms of variability in government misery, we observe an almost perfect reflection of eccentricity variability onto government misery variability. This is notable since parties use office payoffs to decide on government formation too. Nevertheless, the variability of the policy element in government formation is still observable in government misery variability.

Overall, the analyses on variability strengthen our confidence in the results obtained in the mean analysis in the main text.
